# Infarct border-zone biomechanics after myocardial infarction: linking mechanotransduction, fibrosis, and ventricular dysfunction

**DOI:** 10.3389/fbioe.2026.1828513

**Published:** 2026-08-27

**Authors:** Fulufhelo Nemavhola, Thanyani Pandelani

**Affiliations:** 1 Department of Mechanical Engineering, Faculty of Engineering and the Built Environment, Durban University of Technology, Durban, South Africa; 2 Department of Mechanical, Bioresources and Biomedical Engineering, School of Engineering, College of Science Engineering and Technology, University of South Africa, Florida, South Africa

**Keywords:** arrhythmia, cardiac magnetic resonance, fibrosis, finite-element modeling, infarct border zone, mechanobiology, myocardial infarction, remodeling

## Abstract

Myocardial infarction (MI) transforms the left ventricle into a mechanically heterogeneous, evolving composite in which necrotic scar, viable myocardium, edema, microvascular injury, and fibro-inflammatory remodeling coexist. The infarct border zone is the decisive interface of this composite: a region in which surviving myocytes and stressed extracellular matrix (ECM) share load, exchange signals, and progressively reshape one another. Clinically, border-zone phenotype helps explain why patients with similar infarct size diverge toward recovery or progressive remodeling, heart failure, and arrhythmia. Mechanistically, the border zone concentrates “demand” through stress, strain, stress gradients, and shear generated by tethering between contracting remote myocardium and noncontracting or weakly contracting infarct core, by evolving thickness and curvature, and by stiffness gradients that change as edema resolves and collagen networks form and mature. Simultaneously, it determines “capacity” by governing matrix continuity, collagen alignment and cross-linking, and myocyte–ECM coupling that together set stiffness, strength, and tearing resistance, which are not interchangeable and can evolve in different directions. This review synthesizes border-zone biomechanics across scales, integrating histology, echocardiographic strain, cardiac magnetic resonance (CMR) late gadolinium enhancement (LGE) and mapping, diffusion and microstructure imaging, and patient-specific computational cardiomechanics. We connect abnormal border-zone deformation to mechanosensing in myocytes, fibroblasts, endothelial cells, and immune cells via integrins, focal adhesion signaling, stretch-activated channels, cytoskeletal remodeling, and transcriptional regulators including YAP/TAZ and MRTF, and we interpret fibrosis architecture as a mechanically regulated “record” of cumulative loading history. We then evaluate evidence linking border-zone strain patterns, stiffness gradients, microvascular obstruction, and intramyocardial hemorrhage to remodeling trajectory and electrical instability, explicitly distinguishing association, prediction, and causation. Finally, we outline translational requirements for a clinically deployable border-zone risk phenotype that combines imaging with inverse modeling and uncertainty quantification, and we propose testable hypotheses that can be validated in prospective cohorts. A mechanics-first view of the border zone reframes post-MI remodeling as an interface problem and provides a disciplined basis for mechanomodulatory therapies that unload, reinforce, or reprogram the peri-infarct microenvironment.

## 1 Introduction

The contemporary concept of post-infarction remodeling is often narrated as a global story: infarct size, ejection fraction, chamber dilation, and downstream clinical outcomes such as heart failure and sudden death (Pfeffer and Braunwald, 1990; Sutton and Sharpe, 2000; Frangogiannis, 2014). Yet, from the standpoint of mechanics and mechanobiology, remodeling is fundamentally an interface phenomenon that is shaped by spatial gradients in structure, material properties, and activation at the infarct–remote junction (Leancă et al., 2022; Weisman and Healy, 1987). The most decisive region is not the infarct core, which is largely noncontractile and progressively becomes scar, nor the remote myocardium, which may remain structurally intact. Rather, it is the infarct border zone, a transitional band of partially injured, heterogeneously viable tissue that must carry and redistribute load while simultaneously serving as the locus of signaling that drives inflammation, angiogenesis, fibroblast activation, and collagen remodeling (Sutton and Sharpe, 2000; Frangogiannis, 2014; Weisman and Healy, 1987; Travers et al., 2016; Prabhu and Frangogiannis, 2016). The border zone is where surviving myocytes strain against an evolving extracellular matrix; where stiffness gradients generate stress concentrations and shear; and where the mechanical environment is sufficiently abnormal to reprogram both cellular phenotype and tissue microstructure (Leancă et al., 2022; Weisman and Healy, 1987; Guccione et al., 1991).

A mechanics-first definition of the border zone requires distinguishing between demand, capacity, and adaptation. Demand is the mechanically imposed state, described by stress, strain, stress gradients, and shear, and shaped by intracavitary pressure, geometry, fiber architecture, and regional activation (Guccione et al., 1991; Fung, 1991; Humphrey and Rajagopal, 2002). Capacity is the load-bearing ability of the tissue and depends on ECM continuity, collagen alignment and cross-linking, and myocyte–ECM coupling (Prabhu and Frangogiannis, 2016; Leask, 2015). Adaptation is the time-dependent biological response that modifies capacity and, through geometric remodeling and changes in activation, also modifies demand (Pfeffer and Braunwald, 1990; Sutton and Sharpe, 2000; Frangogiannis, 2014; Leancă et al., 2022). This tripartite framing matters because the same border-zone “appearance” may represent different states depending on time after MI and loading conditions. For example, a region may appear “stiff” if edema increases passive resistance to deformation, yet remain mechanically fragile if matrix continuity is compromised (Leancă et al., 2022; Kidambi et al., 2013). Conversely, a region may be compliant but robust if fiber architecture and matrix connectivity distribute load efficiently. Conflating stiffness with strength, or strain with stress, obscures these distinctions and can mislead both mechanistic inference and therapeutic design (Humphrey and Rajagopal, 2002; Holzapfel and Ogden, 2009; Fung and Liu, 1995).

The border zone is also a principal site of electromechanical and mechano-electrical coupling after MI. Surviving myocytes in the peri-infarct region experience altered stretch, altered calcium handling, and altered energetic demand (Bers, 2002; Camelliti et al., 2005). Fibrosis accumulates interstitially and replacement-wise in patterns that can slow conduction, fragment wavefronts, and promote reentry (Camelliti et al., 2005; De Bakker et al., 1993; Spinale, 2007). Mechanical stretch itself modulates electrophysiology through mechanosensitive ion channels and through remodeling of gap junction distribution and cytoskeletal organization (Camelliti et al., 2005; Goldberger et al., 2014; Delmar and Makita, 2012). These couplings help explain why arrhythmia risk is not fully captured by infarct size or left ventricular ejection fraction, and why border-zone heterogeneity, border-zone channels, and substrate features have emerged as important correlates of ventricular tachyarrhythmia and sudden death (De Bakker et al., 1993; Goldberger et al., 2014; Verma et al., 2005; Berruezo et al., 2015; Thomsen et al., 2023).

Clinically, the border zone has been approached through multiple measurement lenses, each reflecting a different facet of demand, capacity, or adaptation. Echocardiographic strain imaging (including speckle tracking) reveals regions of reduced shortening, delayed timing, and mechanical dispersion, while also carrying known sources of inter-platform variability at the segmental level (D’hooge et al., 2000; Nesbitt et al., 2009; Mirea et al., 2018; Gherbesi et al., 2024; Kim et al., 2000). CMR late gadolinium enhancement (LGE) delineates infarct core and “gray-zone” regions of intermediate signal intensity that are often interpreted as heterogeneous tissue, while standardized CMR acquisition recommendations seek to reduce protocol-driven variability in infarct-size quantification, gray-zone classification, and mapping-derived tissue biomarkers (Kim et al., 2000; Rajiah et al., 2013; Kramer et al., 2020; Cerqueira et al., 2002; Schuijf et al., 2004). T1/T2 mapping and extracellular volume (ECV) estimation provide markers of edema and diffuse fibrosis, with consensus recommendations guiding mapping practice and interpretation (Messroghli et al., 2016; Moon et al., 2013; Kammerlander et al., 2016; Han et al., 2023). Markers of microvascular obstruction (MVO) and intramyocardial hemorrhage (IMH) reflect severe reperfusion injury and are linked to contractile recovery and subsequent remodeling trajectories (Kidambi et al., 2013; Turer and Hill, 2010). Diffusion tensor CMR (DT-CMR), where available, provides information on microstructural orientation and disarray after infarction (Nielles-Vallespin et al., 2017; Dall’Armellina and Plein, 2025). Because these modalities do not directly measure stress or strength, a central task of this review is to connect clinical measurements to mechanics and mechanobiology, making explicit what each observation does and does not imply about stress, capacity, and adaptation (Chabiniok et al., 2016; Niederer et al., 2019; Rodero et al., 2023; Pathmanathan and Gray, 2014; Gray and Pathmanathan, 2018; Avazmohammadi et al., 2018).

The review proceeds by first defining the border zone across scales and measurement contexts, emphasizing why differing definitions yield differing estimates and why this matters for translation, including standardized segmentation conventions for imaging-based regionalization (Messroghli et al., 2016). We then examine multiscale heterogeneity in microstructure and material behavior, and we synthesize how stress, strain, and shear emerge and evolve at the infarct–remote interface using contemporary cardiac biomechanics and multiphysics modeling perspectives (Chabiniok et al., 2016; Niederer et al., 2019; Rodero et al., 2023; Pathmanathan and Gray, 2014; Gray and Pathmanathan, 2018; Avazmohammadi et al., 2018). We connect these mechanics to mechanotransduction pathways in myocytes, fibroblasts, endothelial cells, and immune cells—via integrin-based signaling, cytoskeletal remodeling, and YAP/TAZ-linked transcriptional programs—highlighting feedback loops whereby mechanics shapes fibrosis architecture and fibrosis reshapes mechanics (Ingber, 2006; Discher et al., 2005; Dupont et al., 2011; Hinz, 2010; Maeda et al., 2011; Perreault and Black, 2023). We then interpret evidence linking border-zone phenotype to remodeling trajectory, functional recovery, and arrhythmogenic risk, carefully distinguishing association from prediction and prediction from causation (Delmar and Makita, 2012; Verma et al., 2005; Thomsen et al., 2023; Fonseca and Izar, 2022). Finally, we discuss therapy and mechanomodulation, emphasizing trade-offs such as stress redirection and diastolic compliance, and we propose testable hypotheses and validation strategies for a clinically deployable border-zone risk phenotype grounded in verified and reproducible modeling principles (Pathmanathan and Gray, 2014; Gray and Pathmanathan, 2018).

## 2 Methods and evidence acquisition

This article is a structured narrative review rather than a quantitative meta-analysis. No pooled effect-size estimation was undertaken because the relevant literature spans heterogeneous experimental models, imaging modalities, time points after myocardial infarction, species, acquisition protocols, segmentation thresholds, and computational assumptions. The aim was therefore not to statistically aggregate comparable outcomes, but to synthesize mechanistic evidence across biological scales and measurement systems. Evidence was identified through iterative searches of PubMed/MEDLINE and Google Scholar using combinations of terms related to the border zone and its measurement, including “infarct border zone”, “peri-infarct”, “gray zone”, “infarct heterogeneity”, “ventricular remodeling”, “mechanotransduction”, “fibrosis alignment”, “strain imaging”, “speckle tracking”, “feature tracking”, “MR tagging”, “spatial modulation of magnetization”, “late gadolinium enhancement”, “T1 mapping”, “T2 mapping”, “extracellular volume”, “microvascular obstruction”, “intramyocardial hemorrhage”, “diffusion tensor CMR”, “fiber disarray”, “hemodynamic forces”, “haemodynamic forces”, “finite element”, “inverse modeling”, and “patient-specific cardiomechanics” (Kramer et al., 2020; Messroghli et al., 2016; Moon et al., 2013; Chabiniok et al., 2016; Rodero et al., 2023; D’hooge et al., 2000; Nesbitt et al., 2009; Mirea et al., 2018; Gherbesi et al., 2024; Kim et al., 2000; Rajiah et al., 2013; Kramer et al., 2020; Cerqueira et al., 2002; Messroghli et al., 2016; Moon et al., 2013; Nielles-Vallespin et al., 2017; Chabiniok et al., 2016; Niederer et al., 2019; Rodero et al., 2023; Pathmanathan and Gray, 2014; Gray and Pathmanathan, 2018; Avazmohammadi et al., 2018). Searches were not restricted to a single era because conceptual progress spans pre-reperfusion, thrombolysis, and contemporary primary percutaneous coronary intervention periods, and because mechanobiology and tissue-mechanics experiments frequently rely on animal models rather than human cohorts (Turer and Hill, 2010; Galis and Khatri, 2002).

Inclusion emphasized four evidence streams. The first stream comprised foundational clinical and experimental work defining infarct remodeling and infarct expansion and establishing the border-zone interface as the decisive region for load redistribution and progressive dilation, which anchors the demand–capacity–adaptation framing (Pfeffer and Braunwald, 1990; Sutton and Sharpe, 2000; Weisman and Healy, 1987; Fonseca and Izar, 2022; Zardini et al., 1993; Zerhouni et al., 1988). The second stream comprised studies describing infarct healing biology and fibro-inflammatory remodeling, including myofibroblast differentiation, ECM remodeling, and the role of inflammation and mechanoregulation, with emphasis on pathways that plausibly link abnormal mechanics to fibroblast activation and fibrosis architecture (Frangogiannis, 2014; Travers et al., 2016; Prabhu and Frangogiannis, 2016; Hinz, 2010; Bujak and Frangogiannis, 2007; Fomovsky et al., 2012; Li et al., 2025; Francisco et al., 2020; Del Re, 2022; Venugopal et al., 2022; Wells, 2013). The third stream comprised imaging studies that quantify border-zone strain and mechanical dispersion, scar heterogeneity, edema, and microvascular injury, and that relate these phenotypes to remodeling trajectories, arrhythmia substrates, and clinical outcomes, including consensus and standardization documents for CMR mapping and acquisition as well as reproducibility statements for strain measurements. The fourth stream comprised computational and inverse-modeling studies that represent infarct and border-zone mechanics and estimate material properties and stress fields from measured deformation, because such work provides a mechanistic bridge from observable kinematics (strain and displacement) to less directly observable demand and capacity metrics (stress, shear, stiffness, and inferred structural integrity), while also foregrounding identifiability, boundary conditions, and verification/validation as prerequisites for translational inference (Guccione et al., 1991; Chabiniok et al., 2016; Niederer et al., 2019; Rodero et al., 2023; Pathmanathan and Gray, 2014; Gray and Pathmanathan, 2018; Avazmohammadi et al., 2018; Namashiri et al., 2024; Nishimura and Carabello, 2012; Choi et al., 2011; Holzapfel et al., 2000).

Reference lists of key reviews and guidelines were screened to identify additional primary sources, including standardized definitions and regionalization conventions relevant to imaging-based segmentation and cross-study comparability (Kramer et al., 2020; Cerqueira et al., 2002; Messroghli et al., 2016; Moon et al., 2013; Thygesen et al., 2018). Where evidence was inconsistent, we prioritized mechanistic plausibility and explicit discussion of confounders, particularly the dependence of measured deformation on loading conditions and geometry and the dependence of inferred tissue properties on model assumptions and parameter identifiability (Humphrey and Rajagopal, 2002; Pathmanathan and Gray, 2014; Gray and Pathmanathan, 2018; Humphrey, 2008). For imaging studies, particular attention was paid to segmentation approaches (including standardized myocardial segmentation and scar/gray-zone definitions), loading conditions at acquisition, definitions of gray zone and texture/heterogeneity metrics, and outcomes adjudication (Kim et al., 2000; Cerqueira et al., 2002; Gibbs et al., 2018; Moccia et al., 2019). For experimental studies, attention was paid to species, infarct method and size, time after MI, and testing mode and strain rate, including the interpretation of passive mechanical properties in biaxial testing paradigms and regional sampling strategies (Demer and Yin, 1983; Sirry et al., 2016; Ngwangwa et al., 2022; Pandelani et al., 2025; Nemavhola, 2021). For modeling studies, attention was paid to constitutive laws, parameter identifiability, boundary conditions, activation modeling, and validation, consistent with contemporary guidance on patient-specific modeling diversity and verification practice (Pathmanathan and Gray, 2014; Gray and Pathmanathan, 2018; Namashiri et al., 2024). The synthesis distinguishes associations from predictive claims and treats causal statements cautiously unless supported by intervention or perturbation studies (e.g., anti-inflammatory or antifibrotic modulation, or mechanosignaling perturbation) that provide stronger leverage on mechanism (Fonseca and Izar, 2022; Francisco et al., 2020; Venugopal et al., 2022).

To improve transparency regarding the evidentiary basis of this structured narrative review, Table 1 summarizes the major studies and evidence groups included in the synthesis. The table reports study type, species or population, time after MI, measurement modality, operational border-zone definition, primary biomechanical relevance, and reason for inclusion. It is intended to clarify the scope of evidence acquisition and should not be interpreted as a PRISMA meta-analysis dataset or as the basis for pooled quantitative estimation.

**TABLE 1:** Evidence matrix for the structured narrative review.

Evidence stream/ representative source(s)	Study type	Species/ population	Time after MI	Modality/ method	Operational border-zone definition	Primary biomechanical relevance	Reason for inclusion
Foundational ventricular remodeling and infarct expansion studies (Pfeffer and Braunwald, 1990; Sutton and Sharpe, 2000; Weisman and Healy, 1987; Zardini et al., 1993)	Foundational clinical and experimental remodeling literature	Human post-MI populations and experimental models	Acute to chronic	Clinical observation, ventricular geometry, remodeling analysis	Infarct edge and adjacent myocardium considered through infarct expansion, LV dilation, and remodeling trajectory rather than a single imaging-defined border-zone threshold	Establishes the relationship between infarct size, infarct expansion, chamber dilation, wall stress, and adverse remodeling	Included to anchor the demand–capacity–adaptation framework and to explain why the infarct–remote interface is mechanically decisive
Inflammatory healing and post-MI repair biology (Frangogiannis, 2014; Prabhu and Frangogiannis, 2016; Fonseca and Izar, 2022; Phatharajaree et al., 2007)	Mechanistic review and translational biology literature	Experimental MI models and human post-MI evidence	Acute inflammatory phase to chronic repair	Inflammation, immune activation, fibro-inflammatory signaling, healing biology	Infarct and peri-infarct tissue undergoing inflammation, resolution, and reparative fibrosis	Shows how inflammatory signaling, immune-cell activity, and reparative pathways influence scar formation and remodeling	Included to connect border-zone mechanics to inflammation, repair quality, and downstream remodeling
Fibrosis, extracellular matrix remodeling, and cardiac fibroblast activation (Travers et al., 2016; Leask, 2015; Bujak and Frangogiannis, 2007; Venugopal et al., 2022; Hamill and Martinac, 2001; Lyon et al., 2015)	Mechanistic review and experimental evidence	Cardiac fibroblasts, animal MI models, and human cardiac fibrosis literature	Subacute to chronic	ECM remodeling, myofibroblast biology, fibrosis signaling	Fibrotic infarct and peri-infarct regions with interstitial and replacement fibrosis	Explains collagen deposition, matrix remodeling, myofibroblast persistence, stiffness, and diastolic dysfunction	Included to support the distinction between collagen content, collagen architecture, stiffness, and mechanical capacity
Myofibroblast mechanobiology and mechanically regulated collagen architecture (Hinz, 2010; Maeda et al., 2011; Fomovsky et al., 2012; Holzapfel and Ogden, 2003)	Experimental and mechanistic studies	Fibroblast models and healing myocardial infarcts	Subacute healing to scar maturation	Mechanobiology, collagen alignment, ECM mechanics	Healing infarct and border-zone regions exposed to altered mechanical loading	Demonstrates that regional mechanics can influence collagen alignment, matrix remodeling, and myofibroblast-mediated scar organization	Included to support the concept that fibrosis architecture records cumulative mechanical loading history
Cellular mechanotransduction pathways relevant to the border zone (Ingber, 2006; Discher et al., 2005; Dupont et al., 2011; Camelliti et al., 2006; Stewart and Turner, 2021; Simon et al., 2005)	Cell and tissue mechanobiology literature	Myocytes, fibroblasts, endothelial cells, immune cells, and general mechanosensitive cell systems	Applicable across acute, subacute, and chronic phases	Integrins, focal adhesions, cytoskeletal remodeling, stretch-activated channels, YAP/TAZ-linked signaling	Mechanically stressed surviving cells at the infarct–remote interface	Explains how stretch, stiffness, shear, and matrix tension are converted into biochemical and transcriptional responses	Included to support the mechanistic link between abnormal deformation and cellular remodeling
Electromechanical coupling and arrhythmogenic substrate literature (De Bakker et al., 1993; Delmar and Makita, 2012; Verma et al., 2005; Berruezo et al., 2015; Thomsen et al., 2023; Sugden and Clerk, 1998; Ma et al., 2014; Aloysius et al., 2021)	Electrophysiology, imaging, and arrhythmia-substrate studies	Human infarct patients and electrophysiological models	Mostly chronic post-MI	Substrate mapping, LGE-CMR, electrophysiology, mechano-electric feedback	Scar border zone, gray zone, scar channels, slow-conduction corridors, and mechanically stressed peri-infarct myocardium	Links structural heterogeneity, fibrosis, conduction slowing, connexin remodeling, and mechano-electric feedback to arrhythmia risk	Included to explain why border-zone mechanics and fibrosis architecture are relevant to ventricular tachyarrhythmia
LGE-CMR infarct detection, viability, segmentation, and infarct quantification (Kim et al., 2000; Rajiah et al., 2013; Kramer et al., 2020; Cerqueira et al., 2002; Schuijf et al., 2004; Cerqueira, 2002)	Clinical imaging and consensus/guideline literature	Human MI and clinical CMR populations	Acute to chronic	Late gadolinium enhancement CMR and standardized myocardial segmentation	Infarct core defined by hyperenhancement; peri-infarct or gray-zone regions defined by intermediate signal intensity or segmental location adjacent to scar	Provides the imaging basis for infarct-core and peri-infarct tissue classification	Included because LGE remains central to scar-core and gray-zone identification in clinical and research cohorts
LGE gray zone, scar heterogeneity, and texture/granularity phenotypes (Thomsen et al., 2023; Gibbs et al., 2018; Moccia et al., 2019; Unger et al., 2025)	Clinical imaging and risk-stratification studies	Patients with ischemic cardiomyopathy or post-MI scar	Mostly chronic post-MI	LGE-CMR, gray-zone quantification, scar heterogeneity, texture analysis, automated segmentation	Intermediate-intensity tissue, heterogeneous peri-infarct tissue, or channel-like structures adjacent to dense scar	Identifies imaging phenotypes associated with arrhythmia risk and adverse outcomes, while remaining indirect measures of tissue mechanics	Included to support the critical discussion that LGE gray zone is associative and not a direct measure of stress, stiffness, or strength
Parametric mapping and extracellular volume assessment (Messroghli et al., 2016; Moon et al., 2013; Kammerlander et al., 2016; Han et al., 2023; Imanli et al., 2019)	Consensus statements, clinical imaging studies, and tissue-characterization literature	Human CMR populations	Acute to chronic	T1 mapping, T2 mapping, T2* mapping, ECV estimation	Spatial transitions in mapping values around infarcted and peri-infarct tissue	Provides markers of edema, diffuse fibrosis, extracellular-space expansion, and tissue-state evolution	Included to support mapping-based characterization of edema and fibrosis proxies in the border zone
Microvascular obstruction and intramyocardial hemorrhage (Kidambi et al., 2013; Turer and Hill, 2010; Chabiniok et al., 2016; Mendiola et al., 2025)	Clinical CMR and mechanistic reperfusion-injury literature	Reperfused MI and STEMI patients	Acute post-MI to follow-up	CMR MVO, T2/T2*, T1/T2 mapping, contractile recovery and remodeling outcomes	Regions of severe reperfusion injury within or adjacent to the infarct territory	Identifies severe tissue injury, impaired repair, persistent inflammation, and adverse remodeling risk	Included because MVO and IMH modify border-zone mechanics, repair quality, and remodeling trajectory
Diffusion and microstructural imaging of post-MI myocardium (Nielles-Vallespin et al., 2017; Dall’Armellina and Plein, 2025; Davis et al., 2005)	Diffusion CMR and microstructural imaging studies	Human and large-animal post-MI evidence	Acute to chronic, depending on study	DT-CMR, diffusion tensor MRI, microstructural orientation and disarray metrics	Regions of altered fiber orientation, anisotropy, or dispersion near infarct and peri-infarct tissue	Provides evidence that infarction alters fiber architecture and that border-zone microstructure may differ from both scar core and remote myocardium	Included to support the argument that the border zone is a microstructurally distinct region, not merely an intermediate LGE-intensity class
MR tagging and myocardial strain reference methods (D’hooge et al., 2000; Nesbitt et al., 2009; Mirea et al., 2018; Gherbesi et al., 2024; Zerhouni et al., 1988; Eitel et al., 2018; Reindl et al., 2021; Reindl et al., 2019; Xu et al., 2022; Kologrivova et al., 2021)	Imaging-method, reproducibility, and clinical strain literature	Human clinical imaging populations and method-development studies	Acute to chronic, depending on cohort	MR tagging, spatial modulation of magnetization, speckle tracking, CMR feature tracking	Functional border zone defined by reduced strain, delayed shortening, post-systolic shortening, or mechanical dispersion adjacent to infarcted tissue	Establishes strain as a kinematic measure of regional deformation and identifies reproducibility limitations across methods	Included to position MR tagging as a reference-standard strain method and feature tracking/speckle tracking as clinically deployable alternatives
Post-systolic shortening and ischemic timing abnormalities (Ngwangwa and Nemavhola, 2021)	Clinical echocardiography and ischemia literature	Human ischemic heart disease populations	Acute ischemia and post-MI relevance	Echocardiographic strain and timing analysis	Ischemic or infarct-adjacent regions showing delayed contraction or post-systolic shortening	Demonstrates that abnormal timing may indicate tethering, ischemia, altered activation, or mechanical constraint	Included to support interpretation of mechanical dispersion and post-systolic deformation in border-zone assessment
Passive myocardial mechanics and biaxial testing evidence (Demer and Yin, 1983; Sirry et al., 2016; Ngwangwa et al., 2022; Pandelani et al., 2025; Nemavhola, 2021; Takayama et al., 2002; Nemavhola, 2017a)	Experimental mechanics and material-characterization studies	Canine, rat, sheep, pig, and infarcted myocardium models	Healthy, acute, subacute, and chronic states depending on study	Biaxial mechanical testing, regional passive mechanics, material characterization	Tissue regions sampled as infarct, border-zone, remote, or anatomically distinct myocardium	Shows regional anisotropy, nonlinear stiffness, and strain-dependent passive mechanical behavior	Included to ground model assumptions and distinguish deformation, stiffness, strength, and tissue capacity
Constitutive laws and anisotropic soft-tissue modeling (Guccione et al., 1991; Holzapfel and Ogden, 2009; Holzapfel et al., 2000; Mendiola et al., 2024; Calvieri et al., 2023; Fung et al., 1993)	Theoretical and computational biomechanics literature	Soft tissue, myocardium, and cardiovascular tissue models	Not MI-specific but applicable to post-MI modeling	Hyperelastic constitutive modeling, anisotropic fiber-reinforced formulations	Border zone represented through material-property gradients or anisotropic structure	Provides the mathematical basis for representing passive myocardium, anisotropy, and stiffness gradients	Included to support continuum-mechanics interpretation of infarct and border-zone tissue
Ventricular geometry, myofiber architecture, and mechanical heterogeneity (Choi et al., 2011; Sermesant et al., 2006)	Experimental and computational cardiac mechanics studies	Cardiac tissue and ventricular models	Not restricted to MI	Ventricular shape analysis, laminar architecture, myofiber mechanics	Regional myocardium and infarct-adjacent tissue interpreted through geometry and fiber architecture	Shows that ventricular shape and laminar fiber structure influence local mechanical heterogeneity	Included to support the argument that border-zone stress and shear depend on geometry and architecture
Computational cardiomechanics and patient-specific modeling (Chabiniok et al., 2016; Niederer et al., 2019; Rodero et al., 2023; Pathmanathan and Gray, 2014; Gray and Pathmanathan, 2018; Avazmohammadi et al., 2018; Namashiri et al., 2024; Fung, 2013)	Computational modeling, inverse modeling, and data–model fusion literature	Human and animal cardiac datasets	Acute to chronic, depending on application	Finite-element modeling, inverse modeling, electromechanical modeling, data assimilation	Border zone represented by altered material properties, active tension, activation gradients, or inferred stiffness deviations	Enables inference of stress, shear, stiffness, and active tension that cannot be directly measured clinically	Included to bridge observable imaging measures with inferred mechanical demand and capacity
Model credibility, verification, validation, and uncertainty quantification (Niederer et al., 2019; Rodero et al., 2023; Pathmanathan and Gray, 2014; Gray and Pathmanathan, 2018; Imbesi et al., 2025)	Modeling-methodology and computational physiology literature	Computational cardiology and biomedical modeling community	Not MI-specific	Verification, validation, uncertainty quantification, personalization frameworks	Not a biological border-zone definition; applied to border-zone modeling credibility	Establishes that model-derived stress and stiffness estimates are assumption-dependent and require uncertainty reporting	Included to support the manuscript’s emphasis on identifiability, uncertainty-aware modeling, and validation
Hemodynamic force analysis and ventricular flow–wall coupling (Nishimura and Carabello, 2012; Vunjak-Novakovic et al., 2010)	Hemodynamic and clinical imaging/modeling literature	Human STEMI patients and hemodynamic assessment literature	Acute STEMI to follow-up in HDF study	Cine CMR-derived haemodynamic force analysis and hemodynamic assessment	Not a tissue-level border-zone definition; chamber-level force orientation and misalignment	Captures global or chamber-level ventricular flow–wall coupling and may reflect integrated consequences of regional contractile asymmetry, scar tethering, and remodeling	Included in response to reviewer request to discuss HDFs as a chamber-level complement to border-zone mechanics
Remote myocardial contractile adaptation after MI (Nemavhola, 2019b; Sacks et al., 2006; Tesch and Young, 2017)	Clinical, physiological, and computational remodeling literature	Human heart failure populations and post-MI computational/experimental models	Subacute to chronic	Remodeling predictors, hypertrophy physiology, computational prediction of contractile adaptation	Remote myocardium rather than local border zone; included because remote adaptation modifies the load imposed on the infarct–remote interface	Explains that border-zone demand is influenced by remote myocardial compensation, hypertrophy, and altered contractility	Included in response to reviewer request to consider remote myocardium contractile remodeling after MI
Mechanomodulatory and biomaterial intervention literature (Li et al., 2025; Filomena et al., 2022; Mendiola E. A. et al., 2022)	Experimental and translational therapeutic literature	Cardiac repair models and tissue-engineering systems	Mostly subacute to chronic repair	Biomaterials, conductive scaffolds, tissue engineering, microenvironment design	Infarct and peri-infarct tissue targeted for reinforcement, repair, or altered mechanical environment	Tests whether modifying the mechanical microenvironment may influence remodeling and repair	Included to connect border-zone biomechanics to therapeutic design and causal testing
Immune-cell mechanobiology and inflammation-resolution literature (Burlew and Weber, 2002; Ravens, 2003; Zipes et al., 2017; Phatharajaree et al., 2007)	Mechanobiology and immunology literature	Macrophages, immune cells, wound repair, and MI inflammation literature	Acute inflammation to repair phase	Immune mechanobiology, inflammation resolution, metabolic regulation	Inflamed infarct and peri-infarct microenvironment	Supports the idea that mechanical environment can influence immune-cell phenotype and repair trajectory	Included to broaden mechanotransduction beyond myocytes and fibroblasts
Clinical remodeling predictors and multimodal risk phenotypes (Eitel et al., 2018; Reindl et al., 2021; Reindl et al., 2019; Mendiola et al., 2025; Nemavhola, 2019b; Aloysius et al., 2021)	Clinical imaging and outcome studies	STEMI, ischemic cardiomyopathy, and heart failure populations	Acute to chronic follow-up	CMR feature tracking, LGE, biomarkers, multimodality imaging	Infarct, gray zone, scar border zone, and deformation-defined dysfunctional myocardium	Links imaging biomarkers to adverse remodeling, arrhythmia substrate, and clinical outcomes	Included to support translational risk phenotyping and the distinction between association, prediction, and causation

The table summarizes the major studies and evidence groups included in the synthesis, including study type, species or population, time after myocardial infarction, measurement modality, operational border-zone definition, primary biomechanical relevance, and reason for inclusion. The table is provided to improve transparency regarding evidence acquisition and should not be interpreted as a PRISMA meta-analysis dataset or as the basis for pooled quantitative estimation.

**TABLE 2:** Definitions and measurement approaches for the infarct border zone across scales.

Approach	Operational border-zone definition	What it measures most directly	What it does not measure directly	Key assumptions	Common confounders and misclassification
Histology and pathology	Peri-infarct region with mixed viability, inflammation, edema, and evolving fibrosis; often defined by staining transitions or myocyte survival islands	Cellular viability patterns, inflammation, microvascular injury, collagen content and organization at microscopic scale	*In vivo* stress, *in vivo* strain history, active tension; global load at sampling	Sampling represents *in vivo* state; fixation and slicing do not distort structure materially	Spatial undersampling; post-mortem changes; section orientation relative to fibers; difficulty mapping to imaging coordinates
Echo strain (speckle tracking)	Ring or band of reduced strain or delayed mechanical timing adjacent to infarcted segments	Deformation and timing of regional shortening under current loading conditions	Stress, stiffness, collagen alignment, and viability; separates contractility from loading imperfectly	Tracking accuracy and segmentation are adequate; frame rate captures timing; loading is similar across patients	Image quality and noise; heart rate and blood pressure; tethering and translational motion; vendor algorithm differences
CMR LGE	Intermediate signal intensity (“gray zone”) between high-intensity core and normal remote myocardium, using a chosen thresholding method	Relative gadolinium distribution reflecting extracellular space and kinetics at acquisition	Fiber architecture, stiffness, strength; definitive viability at cellular scale; mechanical demand	Thresholds produce consistent tissue classes; spatial resolution sufficient to resolve thin layers	Partial volume, motion, and inversion-time selection; threshold method variability; remodeling changes geometry over time
CMR T1/T2 mapping and ECV	Spatial transitions in T1, T2, or ECV around infarct or within peri-infarct region	Edema-related water content and diffuse fibrosis proxies, depending on sequence and time after MI	Direct collagen alignment and cross-linking; stress; active tension	Sequence calibration stable; hematocrit and field effects corrected; maps reflect tissue rather than artifacts	Heart rate dependence; field inhomogeneity; hematocrit uncertainty; overlap of edema and fibrosis signals early after MI
DT-CMR or microstructure imaging	Regions of altered fiber orientation, reduced fractional anisotropy, or increased dispersion near infarct edge	Microstructural orientation and disarray metrics at voxel scale	Stress and strength; functional loading response	Diffusion encoding captures myofiber orientation despite motion; post-processing robust	Limited availability; long acquisition; resolution and motion sensitivity; interpretation varies with model choice
Computational modeling (forward FE)	Property and activation gradient between infarct and remote myocardium, often informed by imaging intensity transitions	Consequences of assumed heterogeneity for stress, strain, and load transfer	True patient-specific properties unless calibrated; microvascular injury; molecular pathways	Constitutive law and boundary conditions are adequate; assigned gradients represent reality	Parameter sensitivity; non-uniqueness; segmentation uncertainty; neglect of residual stress or pericardial constraint
Inverse modeling and data assimilation	Border zone as region where inferred stiffness or active tension deviates from remote and changes smoothly toward infarct core	Estimated spatial variation in material parameters consistent with observed kinematics and pressures	Microscopic collagen features; unique identification without uncertainty; damage state	Measurements sufficiently informative; regularization does not dominate; priors appropriate	Identifiability limits; pressure estimation error; temporal mismatch; dependence on model form and smoothing

The table contrasts histologic, echocardiographic strain, CMR LGE and mapping, diffusion and microstructure imaging, and computational modeling definitions. Each approach captures different physical or biological quantities and therefore carries distinct assumptions and potential misclassification. Cross-modal translation requires explicit acknowledgment of these differences rather than treating all “border-zone” labels as interchangeable (Kim et al., 2000; Messroghli et al., 2016; Gray and Pathmanathan, 2018; Sermesant et al., 2006).

## 3 Defining the border zone across scales

The term “border zone” is widely used but not uniformly defined, and inconsistent definitions remain a major barrier to translation and cross-cohort comparability (Kramer et al., 2020; Rodero et al., 2023; Cerqueira, 2002). Histologically, the border zone commonly refers to a peri-infarct region in which viable and nonviable myocardium interdigitate, in which inflammation and edema are prominent early, and in which fibrosis develops in replacement and interstitial patterns that may be patchy and directionally organized (Frangogiannis, 2014; Prabhu and Frangogiannis, 2016; Leask, 2015; Kidambi et al., 2013; Venugopal et al., 2022). The spatial extent of this region depends on how viability is defined, on the staining method, and on the sampling strategy, including transmural sampling and regionalization conventions (Frangogiannis, 2014; Prabhu and Frangogiannis, 2016; Cerqueira, 2002). In large-animal and human hearts, the border zone can extend from millimeters to centimeters and can vary circumferentially and transmurally, reflecting coronary anatomy, collateralization, reperfusion injury severity, and transmural perfusion gradients (Frangogiannis, 2014; Kidambi et al., 2013; Benz et al., 2022). The resulting heterogeneity is not merely descriptive: it is the substrate through which scar structure, tethering, and stress transfer emerge as a coupled organ–tissue phenomenon (Fomovsky et al., 2012; Richardson et al., 2015).

Imaging-based definitions often map the border zone to regions with intermediate signal intensity or intermediate functional abnormality. In CMR LGE, infarct core is typically defined by high signal intensity relative to remote myocardium, while “gray zone” is operationalized as intermediate signal intensity between core and remote myocardium (Kim et al., 2000; Kramer et al., 2020; Schuijf et al., 2004; Bogaert et al., 2012). The thresholds used to define gray zone vary widely across studies and platforms, including full-width half-maximum approaches and n-standard-deviation methods, and partial-volume effects and spatial resolution constraints can materially shift the apparent gray-zone extent and its spatial continuity (Kim et al., 2000; Kramer et al., 2020; Schuijf et al., 2004; Bogaert et al., 2012). In addition, LGE reports a contrast distribution shaped by extracellular volume and gadolinium kinetics; it is not a direct map of collagen architecture, collagen cross-linking, or passive mechanical stiffness, and therefore cannot be interpreted as a direct mechanical property field without additional assumptions or corroboration (Rajiah et al., 2013; Kramer et al., 2020; Bogaert et al., 2012). Accordingly, “heterogeneity” metrics derived from LGE texture, granularity, or channel-like morphology should be read as imaging phenotypes that may correlate with arrhythmia substrate or remodeling risk, not as direct measurements of stress, stiffness, or strength (Thomsen et al., 2023; Gibbs et al., 2018; Unger et al., 2025).

Mapping-based definitions use T1, T2, and ECV transitions to characterize edema and diffuse fibrosis, with community recommendations and consensus statements providing guidance on acquisition, quality control, and interpretation (Messroghli et al., 2016; Moon et al., 2013; Kammerlander et al., 2016). These measurements are nevertheless sensitive to sequence choice, heart rate, and physiological state, and ECV estimation depends on hematocrit and methodological standardization, which can confound cross-study comparisons if not controlled or reported (Messroghli et al., 2016; Moon et al., 2013; Kammerlander et al., 2016). Microvascular injury phenotypes such as microvascular obstruction (MVO) and intramyocardial hemorrhage (IMH) further complicate border-zone definition because they reflect severe reperfusion injury that can co-localize with edema and alter regional mechanics and recovery trajectories even when infarct-core size is similar (Kidambi et al., 2013; Turer and Hill, 2010). DT-CMR, where available, provides an additional axis by exposing fiber orientation and disarray, which is directly relevant to anisotropy and stress transfer but is not yet routinely integrated into clinical border-zone definitions (Nielles-Vallespin et al., 2017; Dall’Armellina and Plein, 2025).

Strain-based definitions identify the border zone as a region of reduced shortening, delayed timing, post-systolic features, or increased mechanical dispersion around the scar edge, using echocardiographic speckle tracking or CMR feature tracking approaches (D’hooge et al., 2000; Nesbitt et al., 2009; Mirea et al., 2018; Gherbesi et al., 2024; Eitel et al., 2018; Reindl et al., 2021; Reindl et al., 2019; Xu et al., 2022). Such definitions are attractive because they report function, but they can confound demand and capacity: reduced strain can reflect reduced contractility, increased stiffness, altered activation, or simply altered loading, and similar strain patterns can arise from different stress states in the presence of geometry and boundary-condition differences (Nishimura and Carabello, 2012; Choi et al., 2011). Reproducibility and inter-vendor variability are therefore not technical footnotes but central determinants of whether a strain-defined border zone can serve as a reliable biomarker across sites and cohorts (Mirea et al., 2018; Gherbesi et al., 2024). More fundamentally, because strain is a kinematic outcome, translating strain-defined border zones into statements about stress, shear, or tissue “fragility” requires either additional measurements (e.g., pressure/loading estimates) or model-based inference with explicit assumptions (Humphrey and Rajagopal, 2002; Fung and Liu, 1995; Gray and Pathmanathan, 2018; Masithulela, 2015a; Masithulela F., 2016; Masithulela F. J., 2016; Masithulela, 2015b).

Although echocardiographic speckle tracking and CMR feature tracking are increasingly used because they can be implemented from routine clinical images, CMR myocardial tagging remains the gold-standard reference method for myocardial strain assessment. Spatial modulation of magnetization creates tag lines or grids within the myocardium that can be tracked during the cardiac cycle, allowing direct quantification of regional deformation. This is particularly important when evaluating infarct-adjacent mechanics, where small errors in strain estimation can alter interpretation of border-zone dysfunction, mechanical dispersion, and tethering. However, tagging is limited by additional acquisition requirements, tag fading, spatial-resolution constraints, and post-processing burden. For this reason, tagging is best viewed as the methodological reference against which more deployable strain methods, including CMR feature tracking and echocardiographic speckle tracking, should be interpreted (Mendiola et al., 2025; Mendiola et al., 2024; Mendiola E. et al., 2022)**.**

In computational cardiomechanics, the border zone is often represented as a property gradient between infarct core and remote myocardium, sometimes coupled to an activation gradient representing depressed contractility (Guccione et al., 1991; Chabiniok et al., 2016; Niederer et al., 2019; Rodero et al., 2023; Namashiri et al., 2024; Masithulela, 2015a; Masithulela F., 2016; Masithulela F. J., 2016; Masithulela, 2015b). The gradient width and amplitude are frequently chosen heuristically or derived from imaging-intensity transitions, which is useful for exploring the consequences of heterogeneity and tethering but risks circularity if the model is then used to “explain” stress concentrations that are effectively imposed by the chosen gradients (Chabiniok et al., 2016; Niederer et al., 2019; Rodero et al., 2023; Gray and Pathmanathan, 2018). Conversely, inverse modeling approaches that estimate spatially varying stiffness or active tension from measured deformation can yield data-driven border-zone definitions, but identifiability limits, regularization choices, and verification practices can shape the inferred gradients and the apparent sharpness of the interface (Pathmanathan and Gray, 2014; Gray and Pathmanathan, 2018; Avazmohammadi et al., 2018). These differences matter because border-zone metrics are routinely used to stratify risk and to motivate therapy, and inconsistent definitions can generate apparently conflicting results across cohorts even when the underlying biology is similar (Thomsen et al., 2023; Gibbs et al., 2018; Calvieri et al., 2023).

A practical translation goal is therefore not to choose a single definition, but to build a crosswalk between definitions and to understand how each definition maps to the underlying biomechanical quantities of interest—particularly stress, stress gradients, shear, and capacity proxies such as matrix continuity and anisotropy (Humphrey and Rajagopal, 2002; Holzapfel and Ogden, 2009; Richardson et al., 2015; Fung, 2013; Fung et al., 1993; Gasser et al., 2006). Table 1 summarizes major border-zone definitions and measurement approaches across histology, echocardiographic strain, CMR LGE and mapping, and computational modeling, highlighting key assumptions and common misclassification modes, including segmentation conventions and protocol dependencies that drive cross-study variability (Mirea et al., 2018; Kramer et al., 2020; Messroghli et al., 2016; Gray and Pathmanathan, 2018; Moccia et al., 2019; Cerqueira, 2002). The remainder of the review repeatedly returns to this crosswalk because mechanistic claims about border-zone “heterogeneity” or “stiffness gradients” depend on what was actually measured (or inferred) and on the validity conditions of those measurements and inferences (Pathmanathan and Gray, 2014; Gray and Pathmanathan, 2018; Avazmohammadi et al., 2018).

**FIGURE 1:**
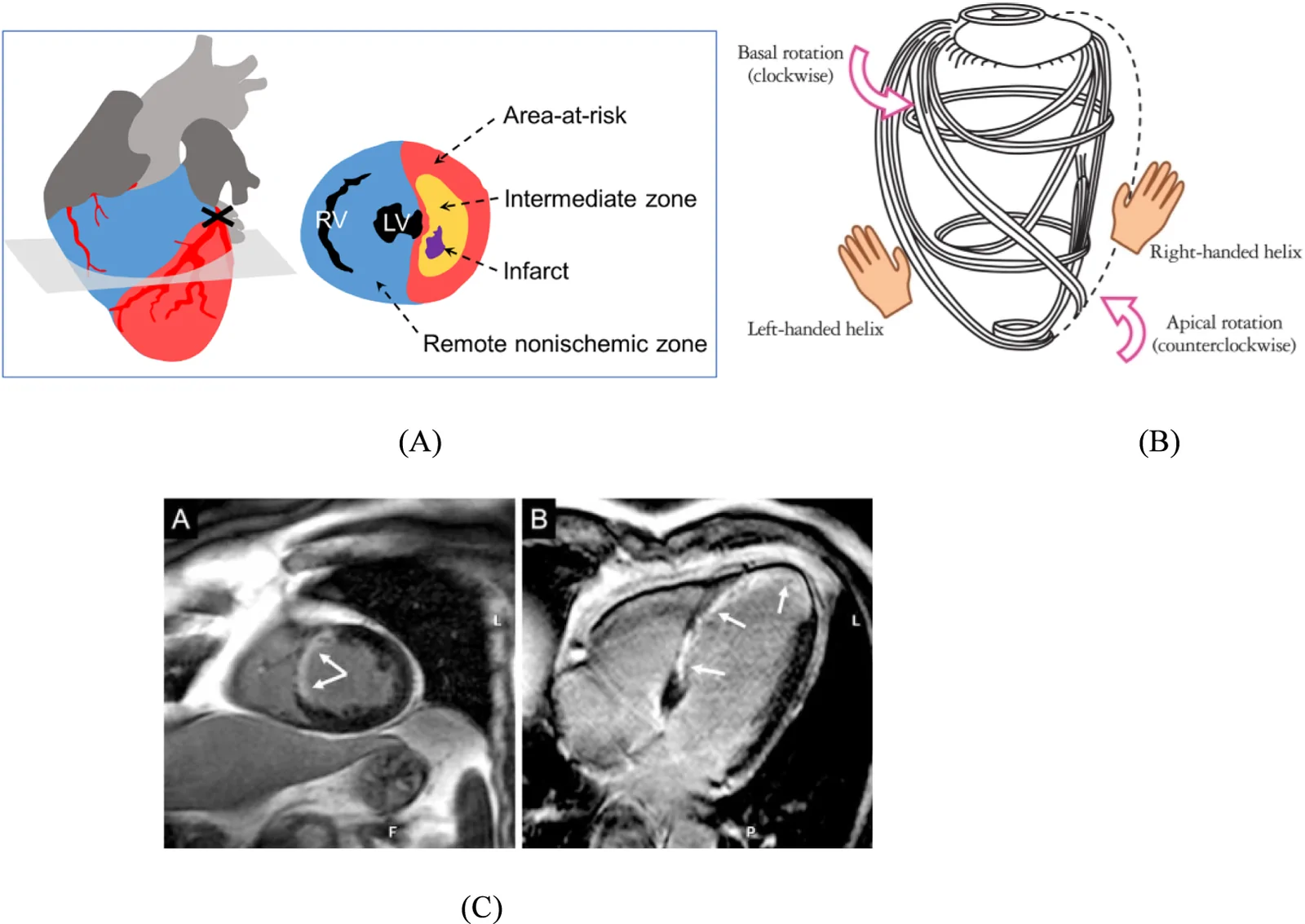
Multiscale structure–function framework of the infarct border zone after myocardial infarction. The schematic integrates processes across biological scales. **(A)** Shows the organ-scale infarct–remote interface, where myocardial infarction produces a scar core surrounded by a mechanically heterogeneous border zone that redistributes ventricular wall stress and strain. **(B)** illustrates ventricular torsion and fiber architecture, emphasizing how altered contraction, twist, and untwist can modify border-zone deformation after MI. **(C)** Shows tissue-level imaging features of infarction and peri-infarct heterogeneity, including scar, gray zone, and adjacent viable myocardium. **(D)** Summarizes the translational framework in which multimodal imaging, strain assessment, microstructural characterization, and patient-specific computational modeling are integrated to quantify regional mechanics and support model-informed risk stratification. The figure emphasizes that the border zone is not simply a transition between scar and remote myocardium, but a multiscale biomechanical interface linking tissue structure, cellular mechanotransduction, ventricular function, and clinical outcomes.

Figure 1 provides a conceptual overview of the infarct border zone as a multiscale biomechanical system that links cellular remodeling to whole-organ ventricular function. Rather than viewing the peri-infarct region as a simple transition between scar and viable myocardium, the Figure 1 synthesizes current evidence showing how spatial heterogeneity in stiffness, fiber architecture, and strain emerges after myocardial infarction and evolves over time. By integrating organ-level mechanics, tissue remodeling, cellular mechanotransduction, advanced imaging, and computational modeling, the illustration establishes the central thesis of this review: that understanding post-MI outcomes requires a unified framework connecting biological scales and translating mechanical insights into clinically interpretable biomarkers and patient-specific risk assessment.

A practical translational goal is not to impose a single universal border-zone definition, but to establish a trial-ready multiscale crosswalk between definitions. In clinical trials, the border zone should be defined prospectively using four aligned layers. The first layer is an anatomical scar reference based on LGE-defined infarct core and peri-infarct gray zone using a prespecified thresholding method. The second layer is a tissue-state modifier based on T1, T2, ECV, microvascular obstruction, and intramyocardial hemorrhage, allowing acute edema-dominated tissue to be distinguished from chronic scar-dominated tissue. The third layer is a functional layer based on co-registered strain, timing, mechanical dispersion, or strain-gradient measures adjacent to the scar boundary. The fourth layer is a model-based mechanical layer in which patient-specific simulations estimate demand indicators such as stress, stress gradients, and shear, while explicitly propagating uncertainty from imaging, segmentation, pressure estimation, and constitutive assumptions. Such a framework would allow histologic, imaging, functional, and computational definitions to be aligned without treating them as interchangeable measurements of the same physical quantity.

## 4 Microstructure and anisotropy in the border zone

The myocardium is an anisotropic composite whose macroscopic mechanics emerge from myocyte fibers, laminar sheet structure, collagen networks, and the coupling between cells and extracellular matrix (ECM) (Humphrey and Rajagopal, 2002; Holzapfel and Ogden, 2009; Camelliti et al., 2005; Fung et al., 1993; Holzapfel and Ogden, 2003; Camelliti et al., 2006). In the intact ventricle, the helix angle of fibers rotates transmurally, supporting efficient torsion, wall thickening, and energetically favourable redistribution of load across the wall (Takayama et al., 2002). Collagen networks provide tensile reinforcement, distribute load between myocytes, and stabilize shear between sheets and laminae, thereby constraining interlaminar sliding and preserving mechanical integrity under multiaxial loading (Humphrey and Rajagopal, 2002; Leask, 2015; Nemavhola, 2021; Fung et al., 1993). Infarction disrupts each component. The infarct core loses viable myocytes and progressively becomes collagen-rich scar with altered orientation and structure, while the border zone is where disruption is incomplete and spatially heterogeneous: islands of surviving myocytes coexist with partially degraded ECM and with newly deposited collagen that is initially disorganized and later becomes more continuous and directionally aligned under load (Fomovsky et al., 2012; Richardson et al., 2015; Fomovsky et al., 2010). The outcome is a region with pronounced directional dependence of stiffness and strong spatial gradients in both passive and active properties, with anisotropy emerging from both microstructural orientation and heterogeneous activation rather than from a single “intermediate” tissue class (Holzapfel and Ogden, 2009; Fomovsky et al., 2012; Fung et al., 1993; Fomovsky et al., 2010).

Microstructural heterogeneity in the border zone manifests across scales. At the cellular level, surviving myocytes undergo cytoskeletal remodeling and altered coupling to ECM through integrin-based adhesions and costameric structures, with mechanosensing pathways integrating altered deformation into transcriptional and phenotypic responses (Ingber, 2006; Discher et al., 2005; Dupont et al., 2011; Maeda et al., 2011; Orr et al., 2006). At the tissue level, myofiber disarray and sheet disorganization can accompany infarct expansion and geometric remodeling, altering the directions along which load is preferentially carried and redistributed (Weisman and Healy, 1987; Zardini et al., 1993; Choi et al., 2011). At the matrix level, collagen deposition is patchy early and becomes more continuous over weeks, with alignment influenced by local principal stretch directions and by myofibroblast traction and mechanoregulated differentiation (Hinz, 2010; Maeda et al., 2011; Fomovsky et al., 2012; Fomovsky et al., 2010). These features contribute not only to anisotropic stiffness, but also to anisotropic failure resistance: an aligned collagen network can carry load effectively along reinforced directions while remaining vulnerable to separation or slip along less reinforced planes, a vulnerability that is amplified when border-zone shear and interlaminar sliding rise at the infarct–remote interface (Humphrey and Rajagopal, 2002; Wells, 2013; Richardson et al., 2015; Fung et al., 1993). In this context, experimental mechanics provides important grounding: multiaxial testing demonstrates that passive myocardium exhibits strong directional dependence under biaxial loading, and post-infarction myocardium can show region-specific shifts in stiffness and nonlinearity that are consistent with evolving collagen architecture and altered microstructural integrity (Demer and Yin, 1983; Sirry et al., 2016; Ngwangwa et al., 2022; Pandelani et al., 2025; Nemavhola, 2017a; Nemavhola, 2019a; Ngwangwa and Nemavhola, 2021; Nemavhola, 2017b; Nemavhola, 2019b).

Diffusion tensor CMR (DT-CMR) and diffusion imaging have provided a noninvasive window into post-infarction microstructure, indicating changes in fiber orientation, helix angle distribution, and measures of orientation dispersion near infarcts (Nielles-Vallespin et al., 2017; Dall’Armellina and Plein, 2025). Where such imaging is available, it supports the concept that the border zone is not simply “intermediate scar,” but a microstructurally distinct region with altered orientation dispersion that plausibly contributes to both mechanical inefficiency and arrhythmogenic substrate (Thomsen et al., 2023; Nielles-Vallespin et al., 2017; Dall’Armellina and Plein, 2025). However, diffusion signals remain indirect and model dependent, and motion, partial volume effects, and spatial resolution limitations complicate interpretation and can blur the transition between scar, border, and remote regions (Nielles-Vallespin et al., 2017; Dall’Armellina and Plein, 2025). Histologic validation remains essential, and a pragmatic translational aim is often not to recover exact fiber vectors in every patient, but to identify robust signatures of disarray and dispersion that add incremental value beyond LGE-defined scar extent and mapping-based measures of tissue composition (Thomsen et al., 2023; Gibbs et al., 2018; Unger et al., 2025).

The importance of diffusion-based microstructural assessment is reinforced by work showing that the post-infarct border zone can be characterized as a distinct microstructural region rather than merely an intermediate imaging intensity class. Kung et al. used diffusion tensor MRI in a post-infarct swine heart model to quantify microstructural remodeling at the infarct border zone and demonstrated that diffusion-derived measures can reveal altered myofiber orientation and tissue organization near the infarct–remote interface [new Kung et al. reference]. This evidence strengthens the concept that the border zone has a measurable microstructural phenotype that may not be fully captured by LGE intensity alone. It also supports the broader argument that border-zone mechanics should be interpreted through anisotropy, fiber disarray, collagen organization, and tissue architecture, rather than through scalar scar burden alone.

Residual stress and prestrain further complicate border-zone mechanics. Even in healthy ventricles, residual stresses can homogenize stress distributions, and prestrain implies that the unloaded configuration does not uniquely determine the *in vivo* stress state (Holzapfel and Ogden, 2009; Fung et al., 1993). After MI, residual stress distributions are expected to change because scar compaction, infarct thinning, and differential growth alter the unloaded configuration, meaning that stress under physiological loading cannot be inferred from geometry alone (Weisman and Healy, 1987; Zardini et al., 1993). Modeling studies that ignore residual stress or prestrain can therefore misestimate border-zone stress gradients, particularly near thin infarct walls or sharp curvature transitions, where small geometric differences can substantially alter inferred demand (Chabiniok et al., 2016; Niederer et al., 2019; Rodero et al., 2023; Pathmanathan and Gray, 2014; Gray and Pathmanathan, 2018; Choi et al., 2011). Although residual stress is difficult to measure clinically, it can be partially accounted for through model calibration to observed deformation and through sensitivity analyses that explicitly acknowledge uncertainty in reference configuration, boundary conditions, and material parameter identifiability (Rodero et al., 2023; Pathmanathan and Gray, 2014; Gray and Pathmanathan, 2018; Avazmohammadi et al., 2018).

A clinically important microstructural dimension is the organization of fibrosis itself. Border-zone fibrosis is not uniform; it includes diffuse interstitial fibrosis that increases passive stiffness and replacement fibrosis that interrupts myocyte continuity and alters cell–cell and cell–matrix coupling (Travers et al., 2016; Leask, 2015; Venugopal et al., 2022). The pattern can be patchy, creating percolating conduction pathways that support reentry and “channel-like” substrate architectures implicated in ventricular tachyarrhythmia, while simultaneously generating mechanical reinforcement in some directions and vulnerability to shear and slip in others (De Bakker et al., 1993; Delmar and Makita, 2012; Dupont et al., 2011; Hinz, 2010). The mechanical implications are therefore inseparable from the electrical implications: fibrosis architecture is both a response to mechanical loading and a modifier of subsequent stress and shear distribution, establishing a feedback loop in which mechanics and microstructure co-evolve (De Bakker et al., 1993; Delmar and Makita, 2012; Dupont et al., 2011; Hinz, 2010). This loop motivates Table 3, which summarizes determinants of stress concentration and shear in the border zone, linking geometry, activation mismatch, microvascular injury, and evolving anisotropy to expected fibrosis architecture.

**TABLE 3:** Determinants of border-zone stress concentration and shear, and expected implications for fibrosis architecture.

Determinant	Biomechanical mechanism in the border zone	Expected effect on stress and shear	Expected effect on fibrosis architecture	Key measurement proxies
Infarct thinning and early expansion	Reduces local thickness and increases local radius, amplifying pressure-driven demand and creating thickness gradients at infarct edges	Increases local stress and stress gradients; increases interface shear due to mismatch in deformation	Promotes aligned reinforcement along principal stretches; may increase replacement fibrosis at highly strained interfaces	CMR wall thickness, regional bulging; echo regional dyskinesia; serial geometry changes
Curvature changes and junction geometry	Creates notch-like regions at transitions between bulging scar and remote wall; concentrates stress at geometric discontinuities	Increases peak stress near junction; localizes shear to border-zone layers	Biases collagen alignment toward junction stress directions; may create patchy reinforcement and residual stress	CMR short-axis curvature indices; 3D shape models; strain gradients near scar edge
Stiffness gradient between infarct and remote tissue	Redistributes load depending on relative stiffness and geometry; can shift stress to stiffer regions and strain to softer regions	Can increase stress concentration at gradient; can increase shear if layers deform differentially	Shapes collagen organization via mechanoregulated deposition; may create anisotropic scar and heterogeneous interstitial fibrosis	Mapping-based ECV gradients; model-inferred stiffness; shear strain estimates from 3D strain imaging
Activation heterogeneity and depressed border-zone contractility	Remote contraction imposes traction on weakly contracting border zone; creates tethering and timing abnormalities	Increases cyclic shear and stress gradients; promotes post-systolic deformation	Promotes mechanosensitive signaling and myofibroblast activation; may reinforce regions with sustained stretch	Echo strain timing and dispersion; CMR feature tracking; electromechanical mapping where available
Microvascular obstruction and intramyocardial hemorrhage	Creates mechanically heterogeneous zones with edema and disrupted matrix; impairs perfusion and repair	Amplifies local heterogeneity and concentrates stress and shear at lesion boundaries	Associated with disorganized remodeling and persistent inflammation, potentially yielding patchy fibrosis and fragile interfaces	CMR MVO on early enhancement; IMH on T2*; T2/T1 mapping signatures
Myofiber and sheet disarray near infarct edge	Alters anisotropic load transmission and increases dispersion of principal strain directions	Increases local shear and nonuniform stress; reduces mechanical efficiency	Promotes heterogeneous collagen alignment and possible conduction heterogeneity via structural discontinuities	DT-CMR dispersion metrics; histology; strain directionality from 3D imaging
Residual stress and scar compaction	Changes unloaded configuration and alters stress distribution under physiological load	Can either homogenize or localize stress depending on distribution; affects border-zone stress gradients	Influences long-term collagen alignment and may stabilize or destabilize interfaces	Model calibration to measured deformation; serial geometry; indirect inference from remodeling patterns

The table summarizes geometric amplifiers, stiffness and activation gradients, microstructural disarray, and microvascular injury signatures. The expected directional effects are qualitative because stress depends on three-dimensional geometry and boundary conditions, but the mapping highlights mechanistically plausible pathways by which deformation patterns could regulate collagen deposition, alignment, and electrical substrate (Sutton and Sharpe, 2000; Holzapfel and Ogden, 2009; Frangogiannis, 2014).

## 5 Border-zone stress, strain, and shear after MI

Regional deformation after MI is dominated by heterogeneity in active tension. The infarct core is noncontractile and may bulge under pressure during systole, while the remote myocardium continues to contract; the border zone must accommodate this mismatch, producing tethering strains and shear at the infarct–remote interface (Sutton and Sharpe, 2000; Weisman and Healy, 1987; Zardini et al., 1993). Even if global ejection fraction is moderately preserved, local border-zone strains can be large in magnitude and temporally abnormal, including delayed shortening, post-systolic shortening, and increased mechanical dispersion around the scar edge (D’hooge et al., 2000; Gherbesi et al., 2024; Asanuma and Nakatani, 2015). These patterns are not merely epiphenomena; they represent the mechanical stimulus that is plausibly most consequential for signaling and matrix remodeling in the region that often determines long-term trajectory (Sutton and Sharpe, 2000; Frangogiannis, 2014; Leask, 2015).

The stress state corresponding to observed strain is not directly measurable, but qualitative scaling clarifies dominant drivers of demand. Pressure loading imposes a baseline stress that increases with chamber radius and decreases with wall thickness, consistent with Laplace-type reasoning and continuum interpretations of wall stress (Humphrey and Rajagopal, 2002; Nishimura and Carabello, 2012; Fung et al., 1993). Infarct expansion and thinning therefore increase demand in and around the infarct, and curvature changes can amplify stress locally, particularly at junctions between bulging infarct and thicker remote tissue (Weisman and Healy, 1987; Zardini et al., 1993; Choi et al., 2011). Stiffness gradients and activation gradients further redistribute load, shifting stress toward stiffer or more strongly activated regions and strain toward more compliant or weakened regions depending on geometry and boundary conditions (Holzapfel and Ogden, 2009; Chabiniok et al., 2016; Choi et al., 2011). This implies that therapies that alter loading (e.g., afterload reduction or mechanical unloading) can meaningfully alter border-zone demand, but it also implies that time-dependent changes in stiffness during healing can redirect stress in ways that may be beneficial or detrimental, emphasizing the need to interpret “stiffening” as a redistribution process rather than a uniformly protective adaptation (Humphrey and Rajagopal, 2002; Wells, 2013; Fomovsky et al., 2010).

Shear is a particularly important component of border-zone demand. In ventricular mechanics, shear arises from fiber–sheet architecture and differential motion between layers, and after MI it can be amplified because remote myocardium attempts to thicken and twist while infarct regions do not (Choi et al., 2011; Takayama et al., 2002). The result is high interlaminar shear and sliding near the interface, which can strain cell–matrix attachments, activate mechanosensors, and promote microstructural remodeling and fibroblast activation (Ingber, 2006; Discher et al., 2005; Dupont et al., 2011; Hinz, 2010; Orr et al., 2006; Stewart and Turner, 2021). Shear also interacts with microvascular injury. Regions with microvascular obstruction (MVO) and intramyocardial hemorrhage (IMH) are mechanically heterogeneous due to edema, hemorrhagic products, and disrupted perfusion, and such heterogeneity can localize deformation gradients and shear (Kidambi et al., 2013). Clinically, MVO and IMH are strong predictors of adverse remodeling and are therefore plausible markers of a border-zone environment prone to maladaptive mechanotransduction, even when infarct size alone is similar (Kidambi et al., 2013; Calvieri et al., 2023; Aimo et al., 2019).

Strain imaging provides an accessible window into border-zone demand, but interpreting strain requires caution. Strain is a deformation metric that depends on loading and on the reference configuration; reduced strain can indicate reduced contractility, increased stiffness, altered loading, or a mixture, and similar strain patterns can arise from different stress states (Humphrey and Rajagopal, 2002; Nishimura and Carabello, 2012). Post-systolic shortening can reflect ischemic segments being stretched in systole and recoiling in early diastole, but can also arise from mechanical interaction with adjacent scar and altered activation timing (D’hooge et al., 2000; Asanuma and Nakatani, 2015). Mechanical dispersion, defined as heterogeneity in timing of peak strain across segments, can reflect electrical dyssynchrony, mechanical tethering, or both; its association with outcomes is therefore mechanistically plausible but not unique, and interpretation benefits from pairing with LGE and mapping to separate tissue composition from timing phenomena (Mirea et al., 2018; Gherbesi et al., 2024; Eitel et al., 2018; Reindl et al., 2021). Disentangling these possibilities requires controlling for infarct size, loading conditions, and comorbidities and, where possible, leveraging standardized acquisition and reproducibility frameworks that reduce methodological variance (Mirea et al., 2018; Kramer et al., 2020; Messroghli et al., 2016).

Patient-specific computational models can infer stress fields from measured deformation and estimated pressure. Finite-element models that incorporate anisotropic constitutive laws and regionally varying active tension can reproduce observed strain patterns and then provide estimates of stress and shear that are not directly measurable by imaging alone (Guccione et al., 1991; Holzapfel and Ogden, 2009; Chabiniok et al., 2016; Niederer et al., 2019; Rodero et al., 2023). However, such inference is sensitive to constitutive assumptions, activation representations, parameter identifiability, and boundary conditions such as basal constraints and pericardial interaction; accordingly, stress estimates should be interpreted as model-based quantities with uncertainty bounds rather than as direct measurements (Rodero et al., 2023; Pathmanathan and Gray, 2014; Gray and Pathmanathan, 2018; Avazmohammadi et al., 2018). A practical translation goal is to identify border-zone stress signatures that are robust across reasonable modeling choices and that correlate with subsequent remodeling, consistent with contemporary emphases on verification, validation, and transparent personalization pathways (Rodero et al., 2023; Pathmanathan and Gray, 2014; Gray and Pathmanathan, 2018; Sacks et al., 2006). Table 3 summarizes mechanical and microstructural determinants of border-zone stress concentration and shear and indicates expected directions of effect on fibrosis architecture, highlighting where predictions are most sensitive to uncertainty and where combined imaging–modeling designs may be most informative (Chabiniok et al., 2016; Rodero et al., 2023; Avazmohammadi et al., 2018).

## 6 Mechanotransduction linking surviving myocytes to fibrosis

Border-zone mechanotransduction is the central link between mechanics and remodeling. Surviving myocytes, fibroblasts, endothelial cells, and immune cells all sense and respond to abnormal deformation, and the border zone amplifies this sensing because stress gradients and shear are often highest at the infarct–remote interface and evolve as scar structure and activation heterogeneity develop (Frangogiannis, 2014; Guccione et al., 1991; Chabiniok et al., 2016; Niederer et al., 2019; Rodero et al., 2023; Richardson et al., 2015). Mechanosensing is mediated by integrin-based adhesions and costameric structures, focal adhesion kinase–linked pathways, cytoskeletal remodeling, and mechanosensitive ion channels that transduce stretch and membrane tension into biochemical signals (Ingber, 2006; Discher et al., 2005; Dupont et al., 2011; Fomovsky et al., 2012; Orr et al., 2006; Stewart and Turner, 2021; Hamill and Martinac, 2001). These pathways converge on transcriptional regulators such as YAP/TAZ and myocardin-related transcription factor (MRTF) and intersect with inflammatory signalling programs, while also interacting with canonical biochemical mediators that coordinate repair and fibrosis, including transforming growth factor-β (TGF-β) signaling and broader profibrotic regulatory networks (Frangogiannis, 2014; Leask, 2015; Dupont et al., 2011; Bujak and Frangogiannis, 2007; Francisco et al., 2020; Del Re, 2022). The net effect is that deformation patterns are translated into gene programs that govern fibroblast activation, collagen deposition, and matrix remodeling, while myocyte hypertrophy and electrical remodeling also respond to stretch and altered load (Bers, 2002; Lyon et al., 2015; Sugden and Clerk, 1998).

Fibroblast-to-myofibroblast differentiation is a key mechanobiological switch. Myofibroblasts generate contractile forces that compact and align collagen, and their persistence contributes to stiffening and diastolic dysfunction (Hinz, 2010; Ma et al., 2014; Burlew and Weber, 2002). Mechanical cues influence this differentiation through substrate stiffness, strain magnitude and timing, and the organization and maturation of adhesion sites (Kim et al., 2000; Discher et al., 2005; Dupont et al., 2011; Perreault and Black, 2023). In the border zone, local stiffness can increase as collagen accumulates, which can promote myofibroblast persistence and establish a positive feedback loop in which stiffness and profibrotic signaling reinforce each other (Leask, 2015; Hinz, 2010; Wells, 2013). TGF-β signaling is often central to this transition, and mechanical activation of latent TGF-β in the matrix provides a direct link between deformation and biochemical signaling, such that high strain and shear environments can amplify profibrotic signaling by converting matrix-bound growth factors into active ligands (Maeda et al., 2011; Bujak and Frangogiannis, 2007). In this framing, border-zone mechanics is not merely an output of remodeling, but an input that shapes fibrosis architecture and its time course (Fomovsky et al., 2012; Wells, 2013).

Surviving myocytes contribute to fibrotic signaling not only through paracrine mediators but also through their own mechanosensitive behavior. Stretch alters calcium handling and energetic demand and can modulate mitochondrial and oxidative pathways that influence inflammatory and fibrotic signaling milieus (Frangogiannis, 2014; Bers, 2002; Turer and Hill, 2010). Mechanical strain can also modulate electrophysiology through mechano-electric feedback and mechanosensitive ion channels, and it can promote remodeling of gap junctional coupling via connexin redistribution, contributing to conduction heterogeneity that evolves alongside structural fibrosis (Delmar and Makita, 2012; Hamill and Martinac, 2001; Ravens, 2003). These changes occur in concert with fibrosis that separates myocytes and increases conduction path length, reinforcing the view of the border zone as a coupled electromechanical system in which deformation influences both matrix remodeling and electrical behavior, thereby linking dysfunction and arrhythmia risk (De Bakker et al., 1993; Delmar and Makita, 2012; Ravens, 2003; Zipes et al., 2017).

Endothelial and immune responses are also mechanosensitive. Shear and strain influence endothelial barrier function and angiogenic signalling, and microvascular injury creates regions of impaired perfusion and hypoxia that can prolong inflammation and alter fibroblast behaviour (Kidambi et al., 2013; Turer and Hill, 2010; Orr et al., 2006). Macrophage phenotypes and cytokine profiles can be co-regulated by mechanical environment, and immune-cell metabolic programs can bias repair toward resolution or persistence of inflammation depending on the microenvironmental constraints (Frangogiannis, 2014; Fonseca and Izar, 2022; Jain et al., 2019; Kologrivova et al., 2021; Aloysius et al., 2021). These interactions support the idea that the mechanical environment is not merely a consequence of injury but a determinant of inflammatory trajectory and repair quality (Frangogiannis, 2014; Prabhu and Frangogiannis, 2016; Fonseca and Izar, 2022). In translation, CMR markers of microvascular obstruction (MVO) and intramyocardial hemorrhage (IMH) can therefore be interpreted as signatures of microenvironments in which mechanotransduction is more likely to be maladaptive because of persistent inflammation, impaired angiogenic recovery, and disrupted matrix continuity (Kidambi et al., 2013; Turer and Hill, 2010; Calvieri et al., 2023).

A key challenge is causal inference. Many mechanotransduction pathways are implicated in fibrosis, but much of the clinical evidence remains associative because direct manipulation of mechanical environment is difficult in humans (Rodero et al., 2023; Gray and Pathmanathan, 2018). Experimental and computational studies provide stronger leverage: altering load, applying mechanical constraint, or changing infarct material environment can modify collagen alignment and scar thickness, implying that mechanics can causally shape fibrosis architecture and subsequent stress redistribution (Chabiniok et al., 2016; Avazmohammadi et al., 2018; Fomovsky et al., 2012). Translating these insights requires acknowledging differences between animal and human healing, infarct size distributions, and pharmacologic backgrounds, and therefore benefits from testable predictions that link specific deformation patterns (particularly gradients and shear proxies) to specific fibrosis architectures and arrhythmogenic substrate patterns (Rodero et al., 2023; Pathmanathan and Gray, 2014).

## 7 Temporal evolution from edema and inflammation to granulation tissue and mature scar

Time after MI is a dominant determinant of border-zone phenotype. Early after MI, the border zone is characterized by edema, inflammatory infiltration, and metabolic stress in surviving myocytes (Frangogiannis, 2014; Prabhu and Frangogiannis, 2016; Turer and Hill, 2010). Edema increases water content and alters relaxation properties and mapping phenotypes, inflammation and proteolysis can weaken matrix continuity, and contractile function is depressed by myocyte injury and disrupted excitation–contraction coupling (Kidambi et al., 2013; Bers, 2002; Spinale, 2007; Messroghli et al., 2016; Phatharajaree et al., 2007). This creates a state in which deformation patterns can be large and heterogeneous and mechanosensitive signaling is intense in the very region that governs load transfer between noncontractile core and functioning remote myocardium (Sutton and Sharpe, 2000; Frangogiannis, 2014; Travers et al., 2016; Ingber, 2006). As days progress, granulation tissue forms, fibroblasts proliferate, and myofibroblasts deposit collagen (Travers et al., 2016; Prabhu and Frangogiannis, 2016; Venugopal et al., 2022). Collagen is initially immature and disorganized, and alignment evolves under local principal stretch directions and myofibroblast traction, consistent with experimentally supported links between regional mechanics and evolving collagen architecture in healing infarcts (Fomovsky et al., 2012). Over weeks, collagen fibers thicken, align, and cross-link, increasing stiffness and likely increasing strength and tearing resistance, while electrical remodeling proceeds on partially overlapping but distinct time scales (Delmar and Makita, 2012; Hinz, 2010; Wells, 2013). Importantly, stiffness can increase before the scar becomes mechanically robust, because collagen content can rise while network continuity and cross-linking remain immature, so stiffness proxies should not be treated as failure-resistance proxies without corroboration (Fomovsky et al., 2012; Wells, 2013; Burlew and Weber, 2002).

The border zone experiences a distinct time course compared with infarct core and remote myocardium. The core progresses toward dense replacement scar with low active tension, while the remote myocardium undergoes hypertrophy and extracellular matrix remodeling in response to altered loading, which can increase diffuse fibrosis and alter diastolic properties (Pfeffer and Braunwald, 1990; Sutton and Sharpe, 2000; Sugden and Clerk, 1998). The border zone sits between these, bearing disproportionate mechanical interactions: early it may be overstretched as remote tissue contracts against a noncontractile region, while later, as the scar stiffens, stress redirection can alter demand in adjacent remote tissue and modify torsion and shear patterns (Zardini et al., 1993; Choi et al., 2011; Takayama et al., 2002). Thus, the border zone is not a fixed ring; it evolves spatially and temporally, and serial imaging is essential to characterize trajectory and time interventions to the evolving mechanical environment (Kramer et al., 2020; Messroghli et al., 2016; Eitel et al., 2018; Reindl et al., 2021).

Recent work on remote myocardial contractile remodeling after MI further supports this interpretation, showing that post-infarction mechanics cannot be understood solely by examining the infarct and border zone, because remote myocardium also undergoes time-dependent changes in contractility, loading response, and mechanical compensation that influence the demand imposed on the infarct–remote interface (Kung et al., 2018; Filomena et al., 2022; Mendiola E. A. et al., 2022).

This temporal evolution also has implications for terminology. An “acute border zone” dominated by edema, inflammation, microvascular injury, and depressed contractility is not mechanically equivalent to a “chronic border zone” dominated by mature scar, collagen cross-linking, persistent stiffness gradients, and stable electromechanical heterogeneity. The spatial extent of the border zone may be wider during the acute phase because edema and inflammatory injury extend beyond the eventual scar boundary, whereas in the chronic phase the border zone may become narrower but mechanically sharper because mature scar and viable myocardium are separated by stronger stiffness, activation, and conduction gradients. Serial imaging is therefore essential, not simply to measure change in infarct size, but to determine whether the same anatomical region is transitioning toward mechanical stabilization, maladaptive stiffening, or persistent demand–capacity mismatch.

Microvascular injury modifies temporal evolution. MVO reflects microcirculatory destruction and is associated with impaired healing and worse outcomes, while IMH reflects severe reperfusion injury and is often observed in association with MVO (Kidambi et al., 2013; Turer and Hill, 2010). These lesions can delay edema resorption, prolong inflammation, and impair collagen maturation, and they introduce sharp heterogeneity in composition and mechanics that can amplify stress gradients and shear (Kidambi et al., 2013; Chabiniok et al., 2016; Calvieri et al., 2023). Therefore, the temporal evolution of the border zone differs meaningfully in MVO/IMH-positive patients, and risk phenotypes should incorporate these markers to avoid pooling fundamentally different healing environments (Kidambi et al., 2013; Calvieri et al., 2023; Aimo et al., 2019).

From a modelling standpoint, temporal evolution motivates time-dependent constitutive and activation parameters. Many computational studies represent infarct stiffness as increasing with time and border-zone activation as partially depressed, while inverse estimation approaches calibrated to serial imaging can, in principle, infer trajectories of stiffness and active tension consistent with observed deformation (Chabiniok et al., 2016; Niederer et al., 2019; Rodero et al., 2023; Avazmohammadi et al., 2018; Namashiri et al., 2024). Identifiability challenges become more acute when parameters evolve, strengthening the case for parsimonious parameterizations that capture dominant changes with a small number of interpretable parameters (for example, a border-zone active tension fraction and a border-zone stiffness ratio relative to remote myocardium, both varying over time) and for verification-focused reporting of model assumptions and uncertainty (Rodero et al., 2023; Pathmanathan and Gray, 2014; Gray and Pathmanathan, 2018).

## 8 Border-zone mechanics as a predictor of remodeling trajectory and functional recovery

A central clinical question is why patients with similar infarct size diverge toward recovery or toward progressive remodeling and heart failure. Border-zone biomechanics offers a mechanistically grounded answer: the border zone governs how load is redistributed after MI, and it is a potent source of mechanosensitive signaling that shapes fibrosis and myocyte adaptation (Sutton and Sharpe, 2000; Frangogiannis, 2014; Fomovsky et al., 2012). Therefore, border-zone phenotype should predict trajectory more strongly than infarct size alone if measured in a way that captures both demand and capacity, and if segmentation and measurement approaches are aligned to the infarct edge rather than diluted across coarse regional averaging (Moccia et al., 2019; Cerqueira, 2002).

Evidence linking abnormal deformation to outcomes is substantial but heterogeneous. Strain reductions and abnormalities in timing are associated with adverse remodeling and events in multiple CMR feature-tracking and echocardiographic strain paradigms, while mechanical dispersion and timing heterogeneity are plausible markers of tethering, activation mismatch, and elevated shear environments (Mirea et al., 2018; Gherbesi et al., 2024; Eitel et al., 2018; Reindl et al., 2021). However, strain is influenced by loading conditions and global function, and it is not a direct measure of stress; accordingly, a key refinement is to focus on gradients and interfaces (border-zone strain gradients and shear-proxy deformation measures) that are more directly linked to tethering and stress concentration than absolute segmental strain values (Gray and Pathmanathan, 2018; Choi et al., 2011; Chitiboi and Axel, 2017). Prospective validation requires consistent segmentation that aligns scar edge with strain measurements, because misalignment can dilute border-zone-specific signals and shift results toward global associations (Mirea et al., 2018; Moccia et al., 2019; Cerqueira, 2002).

LGE-defined gray zone and border-zone channels have been associated with ventricular arrhythmia risk, and scar heterogeneity metrics (including texture and granularity phenotypes) have been reported as outcome-correlates in post-infarction populations (Thomsen et al., 2023; Gibbs et al., 2018; Unger et al., 2025). For remodeling prediction, infarct transmurality and infarct size remain strong predictors, while gray-zone extent and border-zone heterogeneity can provide incremental information in some cohorts but remain method dependent and sensitive to thresholding, resolution, and partial volume effects (Kim et al., 2000; Rajiah et al., 2013; Kramer et al., 2020; Schuijf et al., 2004; Gibbs et al., 2018). Therefore, mechanistic interpretation must remain cautious: a robust observation is that infarct-edge spatial heterogeneity correlates with adverse outcomes, but the mapping from LGE intensity to mechanical capacity is indirect (Rajiah et al., 2013; Rodero et al., 2023; Bogaert et al., 2012). Mapping-based ECV and edema phenotypes provide complementary characterization of diffuse fibrosis and inflammation and can help interpret whether reduced deformation reflects stiffness increases, contractile loss, or microvascular injury–linked dysfunction, particularly when integrated with standardized mapping recommendations (Kidambi et al., 2013; Messroghli et al., 2016; Moon et al., 2013; Kammerlander et al., 2016). MVO and IMH strongly predict adverse remodeling and plausibly represent severe capacity impairment in border-zone microenvironments (Kidambi et al., 2013; Calvieri et al., 2023; Aimo et al., 2019).

## 8.1 Haemodynamic force analysis as a chamber-level complement to border-zone mechanics

Haemodynamic force analysis provides a complementary chamber-level perspective on post-infarction biomechanics. Whereas strain imaging describes myocardial deformation and finite-element modeling estimates wall stress or shear under assumed constitutive and loading conditions, haemodynamic forces describe the net forces exchanged between the intraventricular blood pool and the ventricular walls during the cardiac cycle. These forces can be estimated from cine CMR images using mathematical modeling of ventricular boundary motion, and they can also be studied using advanced flow-imaging approaches. After MI, regional contractile asymmetry, scar tethering, altered chamber geometry, impaired relaxation, and remote myocardial compensation can disturb the normal orientation and timing of intraventricular forces. Such disturbance may reflect an integrated consequence of border-zone dysfunction and altered ventricular flow–wall coupling.

Recent clinical work has shown that intraventricular haemodynamic force misalignment after ST-elevation myocardial infarction is associated with adverse left ventricular remodeling [new Filomena et al. reference]. This suggests that HDF-derived indices may provide early markers of abnormal ventricular mechanics beyond conventional ejection fraction and infarct-size assessment. Importantly, HDFs should not be interpreted as direct measurements of infarct border-zone stress. Rather, they provide a global or chamber-level readout of ventricular flow–wall coupling that may complement LGE, mapping, strain imaging, and patient-specific modeling. A clinically mature border-zone risk phenotype may therefore combine tissue-level measures of scar and gray zone, deformation-based measures of strain gradients and mechanical dispersion, model-derived estimates of stress and shear, and cavity-level HDF metrics reflecting the integrated haemodynamic consequence of regional mechanical dysfunction.

A major translational gap is that many border-zone imaging markers remain associative. LGE gray zone, scar heterogeneity, mapping abnormalities, and strain impairment are clinically informative, but they do not directly prove the presence of a specific stress state, shear field, stiffness gradient, or tissue-failure risk. LGE intensity reflects contrast distribution and extracellular-space expansion; it is not a direct map of collagen architecture, collagen cross-linking, anisotropic stiffness, or mechanical strength. Similarly, reduced strain may reflect impaired contractility, increased stiffness, altered loading, tethering, dyssynchrony, or combinations of these mechanisms. Causal inference therefore requires experimental perturbation, serial observation with mechanistic prediction, mechanomodulatory intervention, or validated computational models that prospectively link a measured imaging phenotype to subsequent remodeling or arrhythmia outcomes. The central translational challenge is to move from the statement that border-zone heterogeneity is associated with risk to the stronger claim that specific border-zone mechanical states predict and mechanistically contribute to risk.

Computational modeling provides a complementary route to prediction by estimating stress fields and quantifying load transfer. If a model calibrated to imaging indicates that border-zone stress is high and border-zone active tension is low, the demand–capacity mismatch is large and mechanistically consistent with progressive remodeling risk (Chabiniok et al., 2016; Niederer et al., 2019; Rodero et al., 2023; Avazmohammadi et al., 2018). The credibility of such inference depends on demonstrating incremental predictive value beyond imaging features alone and robustness to uncertainty in segmentation, pressure estimation, and constitutive assumptions, consistent with contemporary emphases on personalization diversity, verification, and transparent uncertainty handling (Rodero et al., 2023; Pathmanathan and Gray, 2014; Gray and Pathmanathan, 2018). A practical risk phenotype can therefore combine a demand indicator (e.g., a border-zone stress/shear estimate from modeling or a border-zone strain gradient from imaging), a capacity proxy (e.g., ECV/LGE edge phenotypes plus microvascular injury markers), and an adaptation indicator (e.g., serial changes in border-zone deformation or stiffness proxies), with Table 4 mapping candidate biomarkers and modeling outputs to these roles and differentiating association from prediction (Rodero et al., 2023; Pathmanathan and Gray, 2014; Eitel et al., 2018; Reindl et al., 2021).

**TABLE 4:** Candidate border-zone imaging biomarkers and modeling outputs mapped to mechanical meaning and clinical endpoints.

Metric	Primary mechanical role	Plausible mechanistic link	Likely confounders	Clinical endpoints often studied	Validation design needed for prediction
Border-zone strain gradient (echo or CMR feature tracking)	Demand indicator	Higher gradients suggest stronger tethering and stress concentration; likely increases mechanosensing stimulus for fibrosis	Loading conditions, segmentation alignment to scar edge, tracking noise	Adverse remodeling, heart failure hospitalization	Prospective standardized imaging with controlled loading and scar-edge alignment; incremental prediction beyond infarct size
Mechanical dispersion (timing heterogeneity of peak strain)	Demand indicator with electromechanical coupling	Timing heterogeneity implies heterogeneous stress and shear and may correlate with arrhythmogenic substrate	Heart rate, conduction abnormalities, vendor algorithm differences	Arrhythmias, ICD therapies, remodeling	Prospective cohorts with electrical covariates; mechanistic linkage to shear or model-derived stress
LGE-defined gray zone extent	Capacity proxy and substrate marker	Intermediate intensity may reflect heterogeneous fibrosis and surviving myocytes; relates to anisotropic load transfer	Threshold method, partial volume, inversion time selection	Arrhythmias, mortality	Harmonized gray-zone definition and histologic or mapping validation; robust reproducibility
ECV gradient across scar edge (mapping)	Capacity proxy	ECV increases with matrix expansion and fibrosis; gradients may indicate evolving scar–border transition and stiffness gradient	Hematocrit, sequence variability, heart rate dependence	Remodeling, diastolic dysfunction	Standardized mapping protocols; serial studies to link ECV changes to mechanical changes and outcomes
T2 or edema mapping in peri-infarct region	Damage marker and early capacity modifier	Edema alters apparent stiffness and reflects inflammatory milieu that can weaken matrix continuity	Sequence differences, motion, timing after MI	Early remodeling, infarct expansion	Time-resolved studies that separate edema from fibrosis; integration with deformation measures
MVO and IMH presence and extent	Damage marker with capacity implications	Severe microvascular injury disrupts repair, creates heterogeneous mechanics, and predicts maladaptive remodeling	Timing of acquisition, contrast kinetics, T2* sensitivity	Adverse remodeling, heart failure, mortality	Prospective stratification and interaction analyses with mechanical metrics; mechanistic endpoints on healing trajectories
Model-inferred border-zone active tension fraction	Capacity proxy for contractile reserve	Lower active tension increases tethering and stress gradients; reflects surviving myocyte functional integrity	Pressure estimation error, identifiability, model form	Functional recovery, remodeling	Calibration to imaging and pressure with uncertainty bounds; independent validation against functional endpoints
Model-derived border-zone stress or shear index	Demand indicator	Estimated stress and shear integrate geometry, activation, and stiffness gradients; plausible driver of mechanotransduction	Model assumptions, boundary conditions, residual stress	Remodeling, arrhythmias	Benchmarking across modeling pipelines; prospective prediction with uncertainty-aware reporting

Each metric is classified by its hypothesized primary role as a demand indicator, capacity proxy, damage marker, or adaptation indicator, and the table highlights key confounders and the level of evidence needed for predictive translation (Mirea et al., 2018; Kim et al., 2000; Messroghli et al., 2016; Chabiniok et al., 2016).

Haemodynamic force analysis from cine CMR | Cavity-level estimation of the net forces exchanged between the intraventricular blood pool and ventricular walls during the cardiac cycle | Global or regional ventricular flow–wall coupling; orientation and timing of intraventricular force vectors; chamber-level consequence of contractile asymmetry and remodeling | Direct border-zone wall stress, tissue stiffness, collagen architecture, or local mechanical strength | HDF misalignment may indicate abnormal ventricular mechanics and may complement LGE, strain, mapping, and model-derived stress estimates in predicting adverse remodeling | Dependent on image quality, segmentation, mathematical assumptions, temporal resolution, and loading conditions; should be interpreted as a chamber-level complement rather than a direct tissue-level border-zone measurement | Association with adverse remodeling has been reported after STEMI, but prospective validation is required to determine incremental predictive value and mechanistic specificity [new Filomena et al. reference].

**FIGURE 2:**
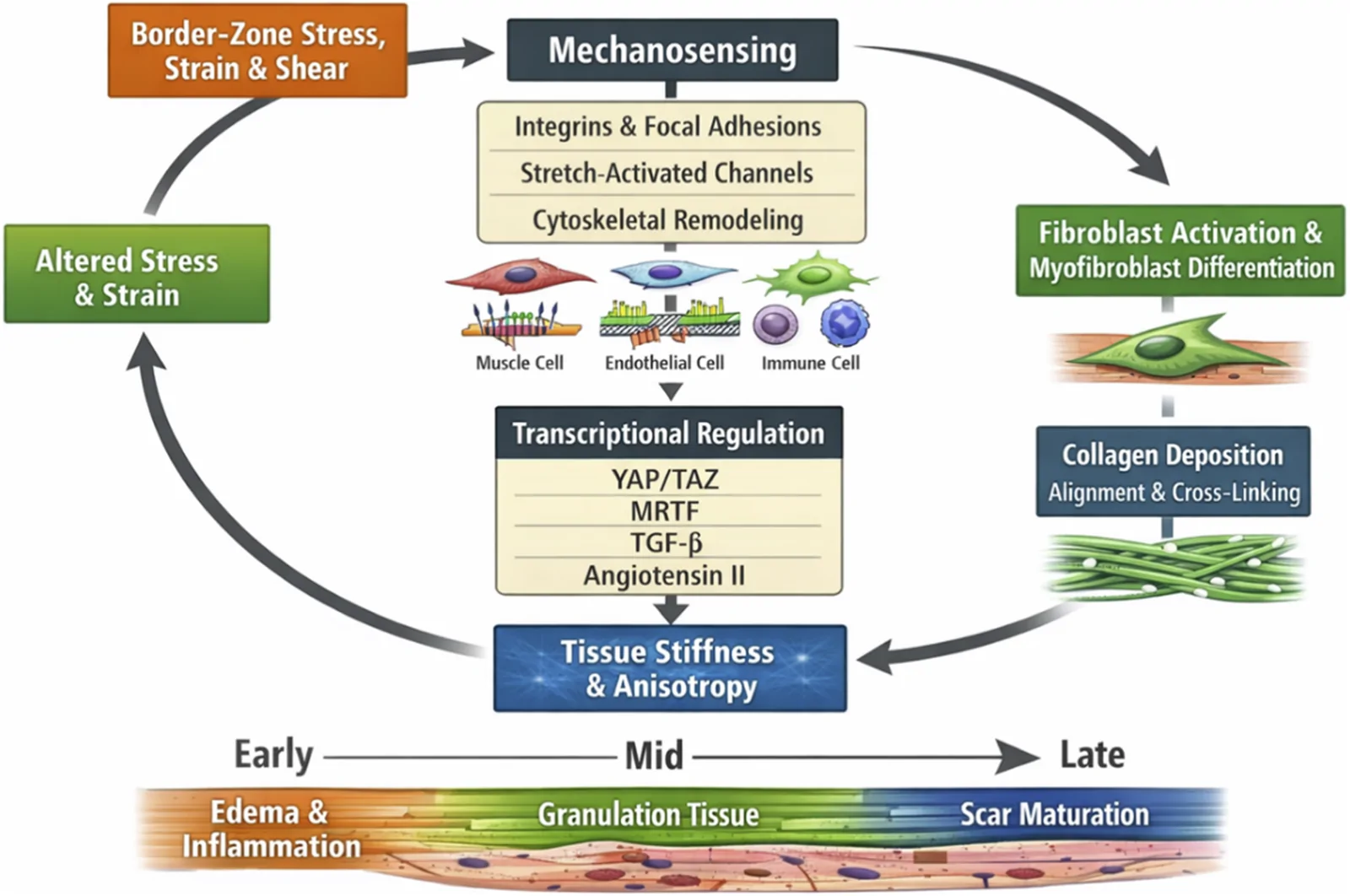
Temporal evolution of the infarct border zone after myocardial infarction. The schematic illustrates the changing spatial extent, tissue composition, and mechanical role of the border zone across three broad phases of healing. In the acute phase, the border zone is dominated by edema, inflammation, microvascular injury, depressed contractility, and mechanically vulnerable matrix disruption. In the subacute phase, granulation tissue, fibroblast activation, myofibroblast differentiation, and early collagen deposition alter local stiffness and begin to stabilize the infarct–remote interface. In the chronic phase, mature scar, collagen cross-linking, anisotropic stiffness gradients, residual stress, and persistent electromechanical heterogeneity define the long-term border-zone phenotype. The figure emphasizes that the border zone is not a fixed geometric ring but a time-dependent interface whose mechanical meaning depends on healing phase, loading conditions, and tissue composition.

Figure 2 provides a mechanistic interpretation for why border-zone deformation patterns are not merely consequences of infarction but plausible drivers of divergent remodeling trajectories. In this framework, stress concentration, strain gradients, and shear at the peri-infarct interface serve as primary mechanical inputs that engage mechanosensing in multiple cell populations—myocytes, fibroblasts, endothelial cells, and immune cells—via integrin-based adhesion signalling, stretch-activated channels, and cytoskeletal reorganisation. These signals couple to transcriptional programs (including YAP/TAZ- and MRTF-linked pathways) and interact with profibrotic mediators such as TGF-β and angiotensin II, promoting fibroblast activation and myofibroblast differentiation. The resulting ECM remodelling is not only an increase in collagen content but also a mechanically patterned reorganisation that includes collagen alignment and cross-linking, thereby altering stiffness and anisotropy in ways that reshape subsequent stress redistribution and deformation—completing a self-reinforcing loop.

A key implication of Figure 2 is temporal specificity: the same intervention (or the same observed imaging phenotype) may have different meanings depending on whether oedema and inflammatory degradation dominate early, granulation tissue is forming in the mid-phase, or scar maturation and cross-linking dominate late. Because electrophysiological remodelling and mechanical remodelling need not proceed on identical time scales, interpreting border-zone fibrosis purely as “good” (structural stabilisation) or “bad” (maladaptive stiffening) is insufficient; instead, the mechanobiology loop argues for timing- and region-aware phenotyping and for trial designs that explicitly test whether reducing mechanical demand can reprogram downstream adaptation.

## 9 Electrical and arrhythmogenic implications of border-zone remodelling

Arrhythmia risk after MI is influenced by both structural substrate and triggering dynamics, and the border zone is central to both (Goldberger et al., 2014; Zipes et al., 2017). Structurally, border-zone fibrosis and surviving myocyte bundles can create slow-conduction channels embedded within scar, supporting reentry, while conduction slowing in healed infarcts is a classic substrate feature that is spatially heterogeneous and interface-dominated (De Bakker et al., 1993; Verma et al., 2005; Berruezo et al., 2015; Thomsen et al., 2023). Functionally, border-zone mechanical stress and stretch can modulate electrophysiology through mechano-electric coupling, influencing action potential dynamics, calcium handling, and ectopy susceptibility (Bers, 2002; Hamill and Martinac, 2001). These interactions provide a mechanistic bridge between border-zone deformation phenotype and arrhythmia risk and motivate integrative phenotypes that treat border-zone remodeling as a coupled electromechanical process rather than separate structural and functional problems (Niederer et al., 2019; Namashiri et al., 2024).

The concept of an LGE “gray zone” has been influential in arrhythmia stratification. Intermediate-intensity regions are interpreted as heterogeneous tissue that can support conduction, and studies relating border-zone mass, channel presence, and heterogeneity phenotypes to ventricular arrhythmia support the value of infarct-edge characterization beyond infarct size alone (Thomsen et al., 2023; Gibbs et al., 2018; Unger et al., 2025). Yet intensity thresholds and spatial resolution can alter gray-zone extent, and causal mapping from LGE signal to conduction properties is indirect (Rajiah et al., 2013; Kramer et al., 2020; Bogaert et al., 2012). A mechanics-first interpretation adds that fibrosis architecture in the gray zone is mechanically regulated and that deformation history may influence channel formation by aligning collagen and promoting patchy deposition, suggesting that combining LGE structure with strain timing/gradient phenotypes may strengthen mechanistic inference compared with either alone (Fomovsky et al., 2012; Eitel et al., 2018; Reindl et al., 2021).

Mechanical stretch itself can be arrhythmogenic. Mechanosensitive ion channels can depolarize myocytes and contribute to premature activity, while heterogeneous stretch can contribute to dispersion phenomena that interact with scar-related conduction heterogeneity (Delmar and Makita, 2012; Hamill and Martinac, 2001). Direct clinical quantification of stretch-triggered arrhythmias is difficult because confounders are substantial, but computational electromechanical models can test hypotheses by coupling deformation fields to electrophysiological dynamics, provided verification and validation practices are explicit and model outputs are treated as uncertainty-bounded inferences (Niederer et al., 2019; Pathmanathan and Gray, 2014; Namashiri et al., 2024). Clinically, substrate mapping and multimodality imaging studies underscore that arrhythmogenic substrate is best approached as an integrated structural–functional phenotype rather than a single-modality feature (Verma et al., 2005; Imanli et al., 2019).

**FIGURE 3:**
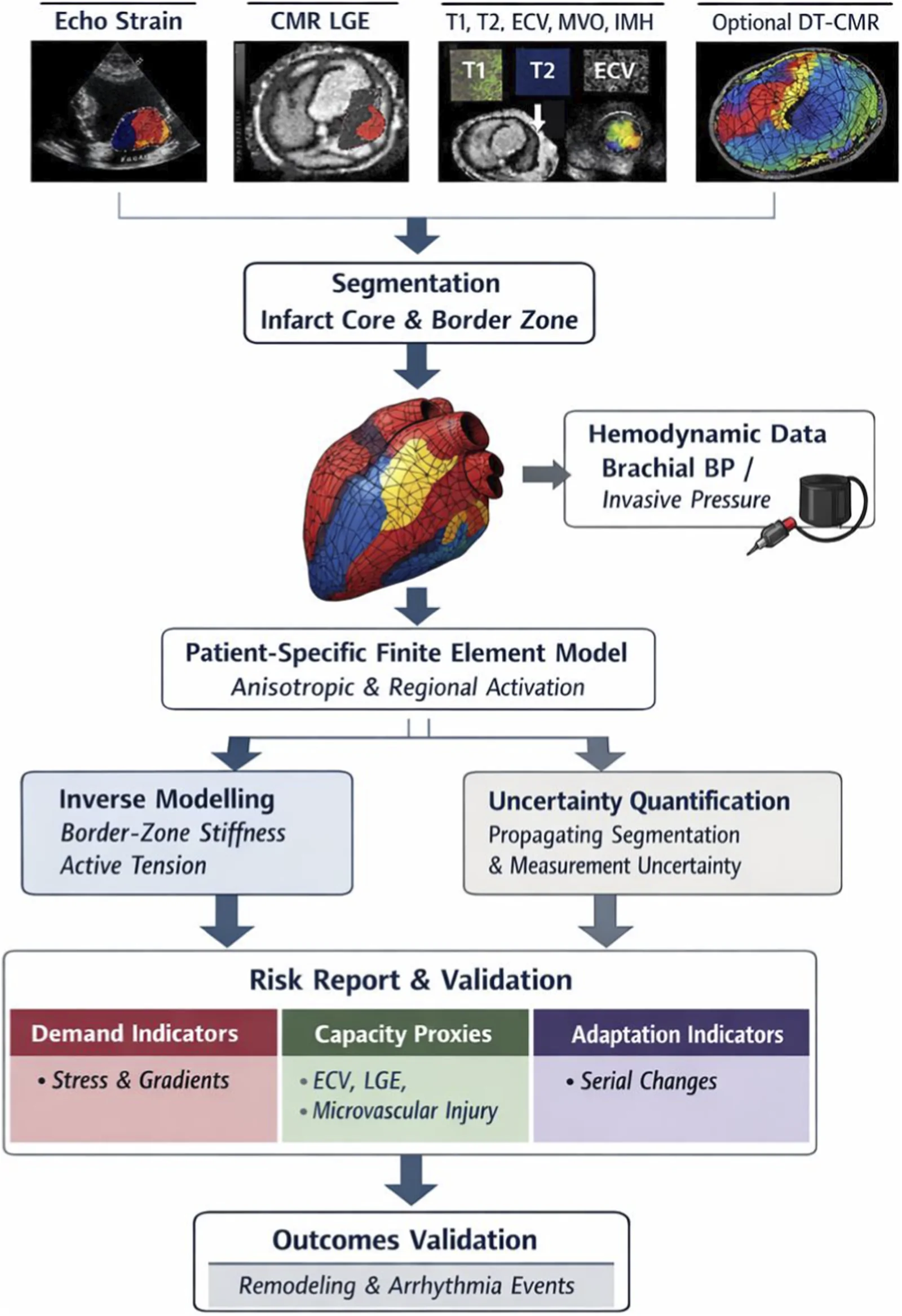
Translational workflow for a clinically deployable border-zone mechanical risk phenotype. Stepwise clinical-to-computational pipeline linking routine imaging to an interpretable, uncertainty-aware border-zone risk output. Imaging inputs include echocardiographic strain and cardiovascular magnetic resonance (CMR) measures such as late gadolinium enhancement (LGE) and mapping (T1, T2, and ECV), alongside microvascular injury markers (MVO and IMH) where available, with optional diffusion-tensor CMR to inform microstructural organisation. The workflow proceeds through segmentation of the infarct core and border zone using a prespecified definition, estimation of loading conditions from brachial (or invasive) pressure, and construction of a patient-specific finite-element model incorporating anisotropic constitutive laws and regionally varying activation. Inverse modelling/data assimilation is then used to estimate border-zone parameters (e.g., effective stiffness and active tension) consistent with the observed deformation. At the same time, uncertainty quantification propagates segmentation and measurement uncertainties into stress, strain-gradient, and shear estimates. The final output is a clinically interpretable report that separates demand indicators (stress, strain gradients, shear), capacity proxies (ECV, LGE edge thickness, microvascular injury), and adaptation indicators (serial changes), and embeds validation checkpoints against remodeling endpoints and arrhythmia outcomes in prospective cohorts, providing a template for mechanomodulatory therapy trials and mechanistic stratification of treatment response.

Figure 3 operationalises the translational argument of this review by showing how border-zone biomechanics can move from an explanatory construct to a deployable clinical phenotype. The workflow links multimodal imaging, strain (echo or CMR feature tracking), CMR LGE and mapping (T1/T2/ECV), and microvascular injury markers (MVO/IMH), with optional microstructural information when available, to a standardised segmentation of infarct core and border zone and to explicit characterisation of loading conditions using contemporaneous hemodynamics. These steps enable patient-specific finite-element modelling with anisotropic material behaviour and regionally varying activation, after which inverse modelling or data assimilation can estimate parsimonious biomechanical parameters (such as border-zone stiffness and active tension fractions) consistent with measured deformation. Critically, uncertainty quantification is positioned as a core requirement rather than an optional add-on, because segmentation variability and measurement noise directly propagate into inferred stress, strain gradients, and shear, which are the quantities most closely linked to mechanobiological stimulus.

By structuring outputs into demand indicators, capacity proxies, and adaptation indicators, Figure 3 also makes the resulting phenotype clinically interpretable and trial-ready. The same pipeline can define inclusion criteria based on a border-zone demand–capacity mismatch, provide mechanistic endpoints (e.g., reduced stress or strain gradients after an intervention), and support mechanistic stratification of treatment response, while embedding validation checkpoints against hard outcomes such as adverse remodeling and ventricular arrhythmias. In this sense, the figure serves as both a risk-phenotyping blueprint and a template for designing mechanomodulatory therapy studies that explicitly test causal hypotheses about border-zone mechanics rather than relying solely on associative imaging markers.

## 10 Implications for therapy and mechanomodulatory interventions

A mechanics-first view of the border zone reframes therapy as modulation of demand, capacity, and adaptation. Many standard therapies align naturally with this framing, even if they are not explicitly designed for mechanics. Early reperfusion reduces infarct size and transmurality and can reduce the magnitude of activation mismatch and tethering, while also reducing the substrate for microvascular injury and inflammatory persistence (Turer and Hill, 2010; Galis and Khatri, 2002; Thygesen et al., 2018). Afterload reduction and hemodynamic optimization reduce pressure-driven stress and thus reduce demand, providing a mechanistic rationale for improved remodeling trajectories under therapies that lower wall stress (Pfeffer and Braunwald, 1990; Sutton and Sharpe, 2000; Nishimura and Carabello, 2012). Neurohumoral modulation and anti-inflammatory strategies can be interpreted as modifying adaptation—specifically, the fibro-inflammatory programs that govern matrix remodeling and myocyte hypertrophy—and emerging approaches to target inflammation after MI highlight the ongoing therapeutic interest in shaping repair quality (Leask, 2015; Fonseca and Izar, 2022; Imbesi et al., 2025).

Mechanical unloading and support devices represent more direct demand reduction. By reducing LV pressure and volume, unloading reduces wall stress and can reduce border-zone deformation, and clinical observations of myocardial recovery under ventricular assist support underscore that load environment can influence functional trajectories, although border-zone-specific microstructural endpoints remain incompletely characterized in contemporary practice (Nishimura and Carabello, 2012; Simon et al., 2005). Because unloading also affects coronary perfusion and systemic physiology, isolating border-zone mechanotransduction effects requires careful study design and multi-parameter phenotyping (Turer and Hill, 2010; Rodero et al., 2023).

Mechanomodulatory biomaterials aim to reinforce infarct and border-zone regions to redistribute stress and reduce injurious deformation. Injectable hydrogels, engineered matrices, and tissue engineering approaches seek to alter local thickness, stiffness, and microenvironmental cues, thereby modulating both mechanics and mechanotransduction (Davis et al., 2005; Vunjak-Novakovic et al., 2010). However, stress redirection is a fundamental trade-off: reinforcing one region can increase stress in adjacent regions, and overly stiff reinforcement can impair diastolic filling and alter torsion and shear patterns (Chabiniok et al., 2016; Choi et al., 2011). Because biomaterial environments can also influence fibroblast behaviour and electrical substrate indirectly through altered strain histories, disciplined design benefits from patient-specific consideration of geometry and infarct location and from modelling that predicts stress redistribution and identifies potential adverse effects (Rodero et al., 2023; Avazmohammadi et al., 2018). Emerging scaffold concepts that explicitly address anisotropy and conduction further illustrate how mechanical and electrical design goals may be coupled in post-infarction repair strategies (Li et al., 2025).

Anti-fibrotic and anti-inflammatory therapies can also be interpreted mechanically. If deformation patterns contribute to profibrotic signalling via mechanotransduction, then targeting mechanosignaling nodes (e.g., YAP/TAZ-linked pathways) or profibrotic mediators (e.g., TGF-β signalling) could decouple demand from maladaptive adaptation. Still, timing is critical because fibrosis is also required for structural stability during early healing (Frangogiannis, 2014; Hinz, 2010; Bujak and Frangogiannis, 2007; Francisco et al., 2020; Del Re, 2022). Therapeutic strategies that alter mineralocorticoid receptor signalling and related fibrosis programs further illustrate the need to treat fibrosis as both a necessary repair and a potential maladaptive stiffening, depending on phase and distribution (Burlew and Weber, 2002; Tesch and Young, 2017). Imaging-informed timing—using serial mapping, LGE phenotypes, strain trajectories, and microvascular injury markers—offers a plausible route to phase-appropriate intervention rather than uniform suppression of repair pathways (Kidambi et al., 2013; Messroghli et al., 2016; Moon et al., 2013; Kammerlander et al., 2016; Eitel et al., 2018; Calvieri et al., 2023).

Revascularization timing and completeness also have mechanical implications. Persistent or recurrent ischemia can sustain border-zone dysfunction and impair recovery of active tension, prolonging tethering and mechanosensitive signaling, whereas restored perfusion can support recovery of contractility and reduce interface mismatch (Galis and Khatri, 2002; Thygesen et al., 2018). This underscores that border-zone mechanics is not purely passive; it evolves with perfusion and metabolic recovery, strengthening the case for integrative imaging strategies that assess structure, composition, and function together when selecting and evaluating interventions (Kramer et al., 2020; Messroghli et al., 2016; Eitel et al., 2018; Aimo et al., 2019).

Figure 3 outlines a translational workflow linking imaging acquisition, segmentation, inverse modeling, and uncertainty quantification to a clinically interpretable border-zone risk output. Such a workflow is also a template for mechanomodulatory therapy trials: it can define inclusion criteria based on border-zone phenotype, provide mechanistic endpoints such as changes in stress or strain gradients, and support mechanistic stratification of treatment response, consistent with contemporary pathways proposed for clinical translation of cardiac biomechanics models (Rodero et al., 2023; Pathmanathan and Gray, 2014; Gray and Pathmanathan, 2018; Moccia et al., 2019).

Uncertainty quantification is essential because inferred border-zone stress, shear, stiffness, and active tension are highly sensitive to upstream measurement and modeling choices. The most important uncertainty sources include infarct-core and gray-zone segmentation thresholds, partial-volume effects, endocardial and epicardial contouring, registration between cine, LGE, mapping, and strain images, estimation of loading conditions from brachial or invasive pressure, assumptions about myocardial thickness and curvature, fiber orientation, pericardial constraint, basal boundary conditions, constitutive-law selection, and regularization strength in inverse modeling. Prospective cohorts should therefore prioritize reproducible scar segmentation, contemporaneous haemodynamic measurements, standardized strain acquisition, co-registration quality control, and sensitivity analyses that report how uncertainty in these inputs propagates into model-derived demand indicators. In clinical translation, a stress or shear estimate should be reported with uncertainty bounds rather than as a single deterministic value.

## 11 Limitations and controversies

Several limitations and controversies constrain border-zone biomechanics as a clinical tool. First, many key quantities of interest are not directly measurable *in vivo*. Stress is an inferred quantity that depends on model structure and boundary conditions, while capacity metrics such as strength, tearing resistance, and interlaminar separation resistance cannot be measured clinically and therefore lack direct patient-level ground truth (Guccione et al., 1991; Chabiniok et al., 2016; Rodero et al., 2023; Pathmanathan and Gray, 2014; Gray and Pathmanathan, 2018). Imaging therefore relies on proxies, but proxies can be misinterpreted if their physical meaning is not explicit; for example, strain is a deformation metric rather than a stress metric, and mapping/LGE primarily report tissue composition proxies rather than collagen architecture or failure resistance (Mirea et al., 2018; Messroghli et al., 2016; Moon et al., 2013). Second, border-zone definitions vary across modalities and across studies. Without harmonized definitions and cross-modal alignment, results can appear inconsistent even when underlying biology is similar (Kim et al., 2000; Kramer et al., 2020; Cerqueira et al., 2002; Moccia et al., 2019; Cerqueira, 2002). Third, loading conditions confound deformation measurements. Blood pressure, heart rate, and volume status substantially alter strain and mapping measurements, and differences across cohorts can masquerade as border-zone phenotype differences unless acquisition context and hemodynamics are measured and controlled (Mirea et al., 2018; Messroghli et al., 2016; Moon et al., 2013; Nishimura and Carabello, 2012). Fourth, the border zone is temporally dynamic. Measurements at a single time point can misclassify state because edema, inflammation, microvascular injury, and collagen maturation evolve rapidly, and the same apparent “intermediate” phenotype can correspond to fundamentally different demand–capacity–adaptation states depending on time after MI (Frangogiannis, 2014; Prabhu and Frangogiannis, 2016; Kidambi et al., 2013; Turer and Hill, 2010). Fifth, computational models face identifiability and validation challenges. Multiple parameter combinations can reproduce similar deformation, and without explicit uncertainty quantification and validation against independent endpoints, model-derived stress metrics can appear more precise than they are (Rodero et al., 2023; Pathmanathan and Gray, 2014; Gray and Pathmanathan, 2018).

A major controversy concerns the meaning of the LGE “gray zone.” While it is widely used as a marker of heterogeneous tissue and linked to arrhythmia risk in several cohorts, intensity-based thresholds are sensitive to acquisition, reconstruction, and choice of reference region, and partial volume effects can generate intermediate intensities even in relatively homogeneous tissue, particularly near thin walls and sharp curvature transitions (Thomsen et al., 2023; Kim et al., 2000; Rajiah et al., 2013). Automated segmentation pipelines can improve reproducibility but do not eliminate dependence on image quality, resolution, and prior assumptions, and therefore can shift apparent gray-zone extent without necessarily reflecting true microstructural heterogeneity (Thomsen et al., 2023; Moccia et al., 2019). Similarly, mapping-based measures (T1/T2/ECV) can be confounded by heart rate, sequence differences, field strength, and hematocrit, so claims that a given mapping phenotype uniquely represents a specific fibrosis architecture should be cautious unless supported by validation or triangulated by complementary measures (Messroghli et al., 2016; Moon et al., 2013; Kammerlander et al., 2016; Han and Zhou, 2022).

Another controversy concerns the directionality and “value” of stiffness changes. Some studies emphasize that scar stiffening is beneficial by limiting infarct expansion and geometric dilation, while others emphasize that stiffening can impair diastolic function and promote stress redirection into adjacent myocardium (Zardini et al., 1993; Fomovsky et al., 2012; Burlew and Weber, 2002). These views are not mutually exclusive; they reflect different time scales and spatial contexts. In early healing, increased stiffness and thickness may stabilize geometry and reduce peak deformation at the interface, whereas in chronic remodeling, excessive diffuse fibrosis and maladaptive matrix remodeling can impair compliance, constrain filling, and interact with electrical remodeling (Leask, 2015; Ma et al., 2014; Burlew and Weber, 2002). The border-zone perspective clarifies that spatially localized reinforcement at the infarct edge may differ mechanistically from diffuse interstitial stiffening across remote myocardium and that timing is central to reconciling apparently contradictory findings (Frangogiannis, 2014; Fomovsky et al., 2012; Ma et al., 2014).

These limitations are especially important when imaging phenotypes are used to infer causality. A gray-zone signal, mapping abnormality, strain reduction, or HDF misalignment may identify a patient at increased risk, but such markers should not be treated as proof of a specific local mechanical mechanism unless supported by convergent evidence. A disciplined translational approach should therefore separate three claims: association, where a marker correlates with remodeling or arrhythmia; prediction, where the marker improves prospective risk stratification beyond established covariates; and causation, where perturbation, serial mechanistic prediction, or validated modeling shows that the measured phenotype contributes to the adverse outcome pathway. This distinction is central to avoiding overinterpretation of border-zone biomarkers.

Finally, causality is a persistent limitation. Many associations between border-zone deformation phenotypes and outcomes may reflect overall disease severity rather than causal pathways. Strengthening inference requires perturbation studies that alter mechanical environment, prospective imaging cohorts with standardized acquisition and hemodynamic context, and integrative models that predict not only stress but also microstructural remodeling patterns that can be validated (for example, collagen alignment signatures or channel-like substrate formation) (Rodero et al., 2023; Pathmanathan and Gray, 2014; Gray and Pathmanathan, 2018; Fomovsky et al., 2012).

## 12 Future directions and testable hypotheses

Border-zone biomechanics offers a set of testable hypotheses that can guide future studies. One hypothesis is that border-zone strain gradients and shear-proxy deformation, rather than absolute strain values, are the primary mechanical stimuli that predict fibrosis architecture and remodeling trajectory. This is motivated by interface mechanics, tethering, and stress concentration arguments and is consistent with the observation that timing abnormalities such as post-systolic shortening often localize near ischemic or mechanically constrained interfaces (Sutton and Sharpe, 2000; Choi et al., 2011; Asanuma and Nakatani, 2015). It can be tested by combining 3D strain imaging (echo or CMR feature tracking) with serial LGE and mapping, explicitly aligning strain fields to the scar edge and quantifying whether gradient-based metrics outperform global strain measures in predicting remodeling endpoints (Messroghli et al., 2016; Moon et al., 2013; Reindl et al., 2021; Reindl et al., 2019). This design should incorporate reproducibility frameworks and segmental alignment standards to avoid conflating true border-zone effects with measurement variance (Mirea et al., 2018; Cerqueira et al., 2002; Cerqueira, 2002) (Kim et al., 2000; Messroghli et al., 2016).

A second hypothesis is that microvascular injury markers identify border-zone microenvironments in which mechanotransduction is maladaptive, such that similar deformation patterns yield worse remodeling in MVO/IMH-positive patients. This can be tested by stratifying cohorts by MVO/IMH and evaluating interaction effects between deformation metrics (particularly gradients or dispersion) and microvascular injury on outcomes, while controlling for infarct size and transmurality (Kidambi et al., 2013; Kim et al., 2000). The mechanistic premise is that MVO/IMH prolongs inflammation and disrupts repair quality, increasing the likelihood that deformation-driven signaling produces adverse fibrosis architecture and persistent heterogeneity (Frangogiannis, 2014; Turer and Hill, 2010; Imbesi et al., 2025).

A third hypothesis is that inverse-model-derived border-zone active tension fraction and stiffness ratio provide a parsimonious, clinically interpretable representation of border-zone demand–capacity mismatch and that these parameters predict outcomes beyond imaging features alone. This hypothesis is directly testable with prospective calibration to measured deformation and pressure estimates and with uncertainty quantification that reports credible intervals rather than point estimates (Chabiniok et al., 2016; Rodero et al., 2023; Pathmanathan and Gray, 2014; Gray and Pathmanathan, 2018; Avazmohammadi et al., 2018). The design requirement is rigorous: the model must be verified, parameter identifiability must be addressed (including sensitivity to boundary conditions and constitutive choices), and predictive claims should be evaluated against endpoints not used in fitting (Niederer et al., 2019; Pathmanathan and Gray, 2014; Gray and Pathmanathan, 2018).

A fourth hypothesis is that fibrosis alignment in the border zone reflects the directionality of principal stretches during healing and that alignment signatures predict both mechanical dysfunction and arrhythmogenic channel formation. The mechanistic basis is that regional mechanics influences collagen structure during healing, and collagen alignment is reinforced by myofibroblast traction in a mechanically regulated microenvironment (Hinz, 2010; Fomovsky et al., 2012). Testing this hypothesis requires either microstructure imaging (DT-CMR where available) or targeted histologic validation in selected cohorts, integrated with electrophysiological substrate characterization, including evidence on channel-like conduction corridors and border-zone channels associated with ventricular arrhythmia (Verma et al., 2005; Berruezo et al., 2015; Thomsen et al., 2023; Nielles-Vallespin et al., 2017; Dall’Armellina and Plein, 2025). Because conduction heterogeneity is also influenced by connexin remodeling and mechano-electric feedback, integrative designs should consider coupling structure, deformation, and electrophysiology rather than treating these domains independently (Delmar and Makita, 2012; Hamill and Martinac, 2001).

Methodologically, progress depends on standardization. Cross-vendor standardization of strain, harmonization of LGE gray-zone definitions, and consensus mapping acquisition are prerequisites for multi-center studies, particularly if gradient-based metrics are to be compared across sites (Mirea et al., 2018; Kramer et al., 2020; Messroghli et al., 2016; Moon et al., 2013). Standardized segmentation frameworks also matter because spatial misregistration between scar edge and strain/mapping is a dominant error source when attempting border-zone-localized inference (Cerqueira, 2002). For modeling, open benchmarking datasets that include imaging, pressure estimates, and outcomes would enable comparative validation of pipelines and uncertainty quantification methods, aligning with broader calls for verification, validation, and transparent personalization pathways in computational cardiology and physiome-aligned science (Niederer et al., 2019; Pathmanathan and Gray, 2014; Gray and Pathmanathan, 2018; Crampin et al., 2004).

Finally, mechanomodulatory interventions provide a route to causality. Trials of biomaterial reinforcement, scaffold-based repair, or unloading strategies can incorporate border-zone mechanical phenotypes as inclusion criteria and mechanistic endpoints, using workflows that quantify whether interventions reduce demand (stress/strain gradients and shear proxies) or reprogram adaptation (fibrosis architecture trajectories) without unacceptable trade-offs in diastolic function or arrhythmia risk (Rodero et al., 2023; Li et al., 2025; Davis et al., 2005; Vunjak-Novakovic et al., 2010; Schwartz and DeSimone, 2008). The value of such trials is not only clinical efficacy, but mechanistic discrimination: if changing the mechanical environment shifts remodeling trajectories in ways predicted by mechanotransduction logic, the border-zone biomechanics framework moves from plausible association to actionable causality (Discher et al., 2005; Dupont et al., 2011; Fomovsky et al., 2012; Orr et al., 2006).

## 13 Conclusion

The infarct border zone is the mechanically decisive interface of the healing ventricle. It concentrates mechanical demand through stress, strain gradients, and shear generated by tethering and evolving geometry, and it governs mechanical capacity through evolving matrix continuity, collagen alignment, and myocyte–ECM coupling. Because border-zone mechanics drives mechanotransduction in myocytes, fibroblasts, endothelial cells, and immune cells, it links surviving myocytes to fibrosis architecture and to whole-ventricle dysfunction. Imaging modalities provide complementary windows into this region but capture different quantities and can be confounded by definitions and loading. Patient-specific modeling can infer stress and capacity surrogates but must be uncertainty-aware and validated. A mechanics-first border-zone phenotype that combines deformation gradients, microvascular injury markers, and structural proxies for fibrosis and edema offers a promising route to explaining divergent trajectories after MI and to guiding mechanomodulatory interventions. The most realistic next steps are standardized, prospective, multi-modal imaging cohorts paired with calibrated modeling and outcome validation, designed explicitly to test mechanistic hypotheses about demand–capacity–adaptation feedbacks in the peri-infarct region.

Border-zone biomarkers and model outputs should be interpreted according to whether they primarily reflect demand, capacity, damage, or adaptation. Demand indicators are those that report deformation heterogeneity, strain gradients, shear, or inferred stress. Capacity proxies are those that report matrix expansion and fibrosis burden or maturation, such as ECV or LGE edge thickness, and microvascular injury signatures that indicate impaired repair. Damage indicators capture microvascular injury and edema that suggest disrupted microstructure and persistent inflammation. Adaptation indicators capture serial changes that indicate whether the system is stabilizing or progressing. Table 4 maps commonly used candidate biomarkers to these roles and highlights likely confounders and validation needs.
